# Identification and validation of methylated differentially expressed miRNAs and immune infiltrate profile in EBV-associated gastric cancer

**DOI:** 10.1186/s13148-020-00989-0

**Published:** 2021-01-29

**Authors:** Mansheng Zhu, Qixiang Liang, Tao Chen, Qian Kong, Gengtai Ye, Shitong Yu, Xunjun Li, Qinglie He, Hao Liu, Yanfeng Hu, Jiang Yu, Guoxin Li

**Affiliations:** 1 Department of General Surgery, Nanfang Hospital, Southern Medical University, 1838 North Guangzhou Avenue, Guangzhou, 510515 China; 2 Department of Stomatology, The Third Affiliated Hospital of Sun Yat-Sen University, Guangzhou, 510630 China; 3 Department of Pediatrics, The Third Affiliated Hospital of Sun Yat-Sen University, Guangzhou, 510630 China

**Keywords:** DNA methylation, miRNA, Gastric cancer, Integrative analysis

## Abstract

**Background:**

The recent discovery of cancer/tissue specificity of miRNA has indicated its great potential as a therapeutic target. In Epstein–Barr virus-associated gastric cancer (EBVaGC), host genes are affected by extensive DNA methylation, including miRNAs. However, the role of methylated miRNA in the development of EBVaGC and immune cell infiltration has largely remained elusive.

**Results:**

After crossmatching the DNA methylation and expression profile of miRNA and mRNA in the Gene Expression Omnibus (GEO) and the Cancer Genome Atlas Research Network (TCGA), we discovered that miR-129-2-3p was significantly suppressed due to hypermethylation on its enhancer in EBVaGC. The differentially expressed genes (DEGs) added up to 30, among which AKAP12 and LARP6 were predicted to be the target genes of miR-129-2-3p and negatively correlated with patients’ survival. Accordingly, miR-129-2-3p was significantly down-regulated in tumor samples in 26 (65%) out of 40 cases in our cohort (*P* < 0.0001). The proliferation, migration and invasion functions of GC cells were significantly promoted when transfected with miR-129-2-3p inhibitor and suppressed when transfected with mimics or treated with 5-aza-2′-deoxycytidine. Moreover, a comprehensive regulation network was established by combining the putative transcription factors, miRNA-mRNA and protein–protein interaction (PPI) analysis. Pathway enrichment analysis showed that cytokine activity, especially CCL20, was the most prominent biological process in EBVaGC development. Immune cell infiltration analysis demonstrated CD4^+^ T cell, macrophage and dendritic cell infiltrates were significantly enriched for the prognostic-indicated hub genes.

**Conclusion:**

This study has provided a comprehensive analysis of differentially expressed miRNAs and mRNAs associated with genome-wide DNA methylation by integrating multi-source data including transcriptome, methylome and clinical data from GEO and TCGA, QPCR of tumor samples and cell function assays. It also gives a hint on the relationships between methylated miRNA, DEGs and the immune infiltration. Further experimental and clinical investigations are warranted to explore the underlying mechanism and validate our findings.

## Background

Gastric cancer (GC) is the second most common cancer in China, accounting for nearly 500,000 cancer deaths annually [1]. The dire prognosis of GC is partly due to its heterogeneity. In 2014, the Cancer Genome Atlas Research Network (TCGA) has revealed the 4 subtypes of GC, tumors positive for Epstein–Barr virus (EBV), microsatellite unstable tumors, genomically stable tumors and tumors with chromosomal instability [2]. The EBV-associated gastric cancers (EBVaGC) are particularly attracting much attention recently, since they are often correlated with increased lymphocytic infiltration with high expression of PD-L1, demonstrating higher sensitivity to immune checkpoint therapies [3]. Although it has been reported that EBVaGCs are abundant with DNA methylation, little is known of the role of EBV infection during the development of GC and its effect on the efficacy of immune checkpoint therapy.

Epigenetic alterations are reversible and have immense therapeutic potential. They could be seen as surrogate markers for exposure to environmental factors, such as infections and hypoxia. DNA methylation, among others, is of peculiar importance in the process of gastric carcinogenesis in that cancer-related genes are more frequently methylated than mutated [4]. Aberrant DNA methylations start accumulating at the early onset of gastric carcinogenesis [5]. The distinct methylation status of certain genes is correlated with immune response, tumor metastasis and location [6, 7]. RUNX3 hypomethylation, for instance, was found to be a biomarker for early GC detection and premalignant immune involvement [8], while FLI1 hypermethylation in tissue and plasma samples was associated with liver metastasis [9]. EBVaGC, in particular, possesses the highest level of DNA methylation in solid tumors [2] and that its objective response rate (ORR) to PD-1 inhibitor is significantly higher than EBVnGC [10, 11], indicating a unique role of EBV-induced methylation in GC development and treatment response. Since the heterogeneity of immune infiltration might be a trait intrinsic of tumor cells [12], epigenetic changes such as DNA methylation could serve as a dynamic marker for the immune infiltration profile.

Unlike the chronic inflammatory intermediate pathways induced by H. pylori infection, EBV is capable of directly modulating host DNA methyltransferase (DNMT1) and down-regulating TET2 demethylase to arouse extensive methylations [13, 14]. In EBVaGC, the methylated sites largely locate at the CpG island in the promoter regions, which is termed CIMP (CpG island methylator phenotype), thus affecting the downstream signaling pathways to regulate cell cycle, apoptosis, cell migration and invasion [15]. Additionally, methylations could also take place at other regions, such as the enhancers. Due to the binary methylation status of cytosines, these CpG-poor regions tend to have fairly variable methylation [16]. Recently, researchers discovered that EBV infection could induce aberrant alterations in enhancers and promote carcinogenesis and aggressiveness [17].

MicroRNAs (miRNAs) are small non-coding single stranded RNAs with ~ 22 nucleotides in length, which function post-transcriptionally as negative regulators by binding to the 3′-untranslated regions (3′-UTR) of target mRNAs. Multiple studies suggested that miRNAs were specifically expressed in certain types of tumors or tissues [18, 19], encompassing a huge potential for precise targeting. In EBVaGC, miRNAs could derive both from EBV per se and the host genome. The EBV-encoded miR-BARTs, for instance, was reported to repress apoptosis and induce dedifferentiation [20], while host miR-200, miR-143-3p and miR-146b were found to be down-regulated by EBV infection and enhanced metastatic potential of the tumor cells [21]. Epigenetic modifications like DNA methylation could be carried out via miRNAs to mediate alterations in chromatin regulatory regions [22]. However, the impact of DNA methylation of miRNA during the development of EBVaGC is seldom discussed.

In the present study, miRNA and mRNA sequencing and methylation profiling data from the TCGA database of stomach adenocarcinoma (TCGA-STAD) as well as multiple microarray datasets from the Gene Expression Omnibus (GEO) database were used for bioinformatic analysis, together with cellular assays, to delineate the possible regulatory network in EBVaGC, including miRNA-mRNA, PPI, and pathway enrichment analysis. Hub genes were also evaluated for their prognostic indications. Moreover, immune cell infiltration profile was investigated to illustrate the specific immune microenvironment of EBVaGC that held higher response rate to PD-1 inhibitor.

## Results

### Identification of methylated differentially expressed miRNAs in EBVaGC

**Fig. 1:**
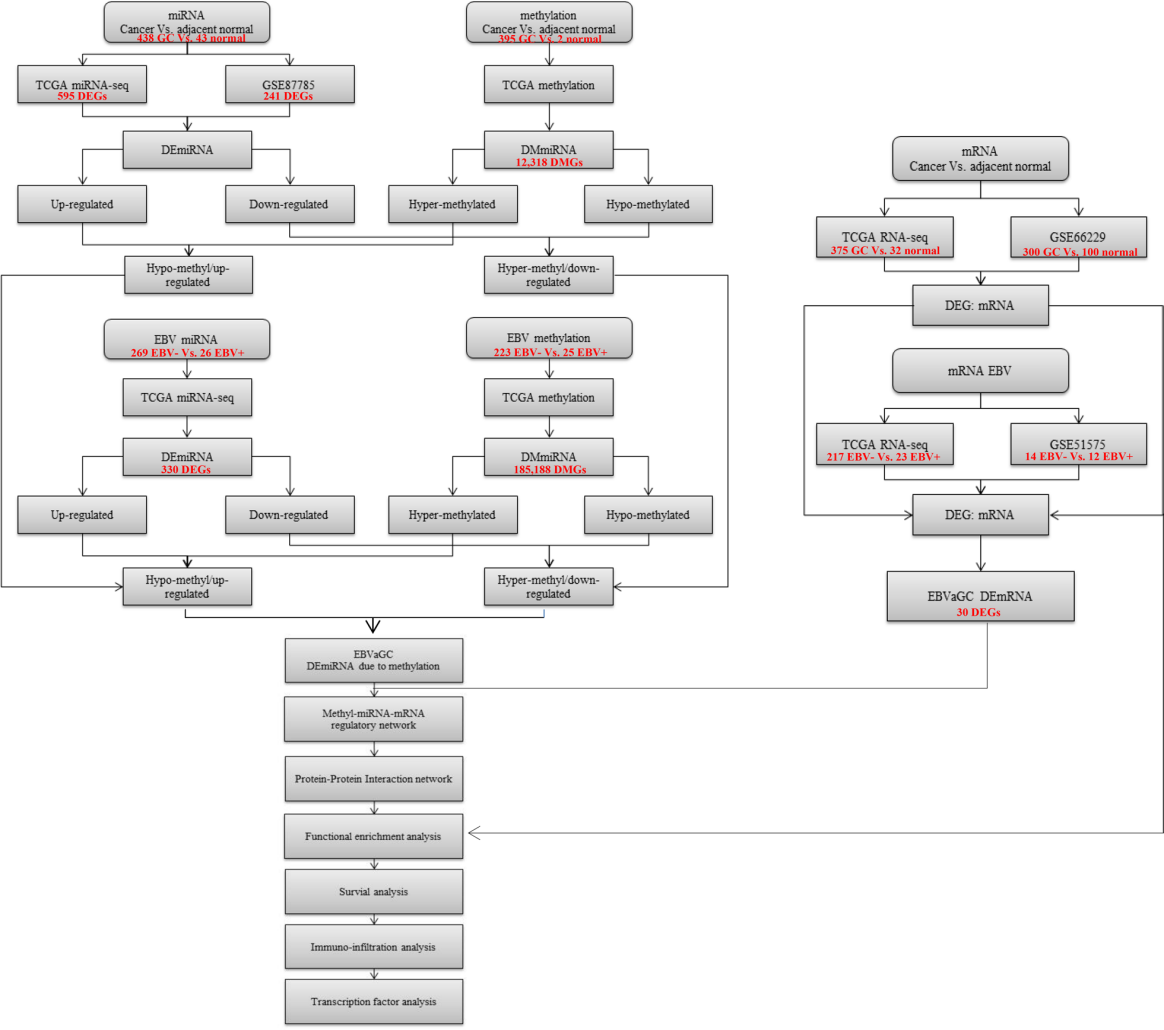
The workflow of the present study. RNA-seq and methylation-seq data from the TCGA-STAD and GEO datasets were extracted and analyzed. The key miRNAs and mRNAs were discovered by crossmatching the differentially expressed methylated miRNAs and differentially expressed mRNAs in EBVaGC. Subsequently, a comprehensive regulation network was established using a series of online analyzing tools

The procedure for data analysis was compiled into a flowchart (Fig. 1). As was shown in the flowchart, after cross-matching the results of all the globally differentially expressed miRNAs, there were 5 miRNAs whose expression was suppressed due to hypermethylation, while none with high abundance due to hypomethylation (Additional file 1: Fig. S1a). TCGA-STAD miRNA-seq datasets with information of EBV infection and methylation were also extracted, followed by probe annotations of differentially methylated sites and identification of the corresponding miRNA accordingly. Cross-matching the results of EBV related DEmiRNAs and methylation analysis, there were 69 down-regulated miRNAs with hypermethylation, while there were 2 miRNAs with high abundance and hypomethylation (Additional file 1: Fig. S1a). To further delineate the DEmiRNAs in EBVaGC due to methylation, we again integrated the results of MDEGs from GC and EBV datasets and found miR-129-2-3p to be the only one gene that was down-regulated due to hypermethylation in EBVaGC (Additional file 1: Fig. S1a). In the meantime, TCGA datasets as well as GSE51575 and GSE66229 from GEO were used to find out the DEGs in EBVaGC (Additional file 1: Fig. S1b). After the crossmatch, 30 DEGs were found.

**Fig. 2:**
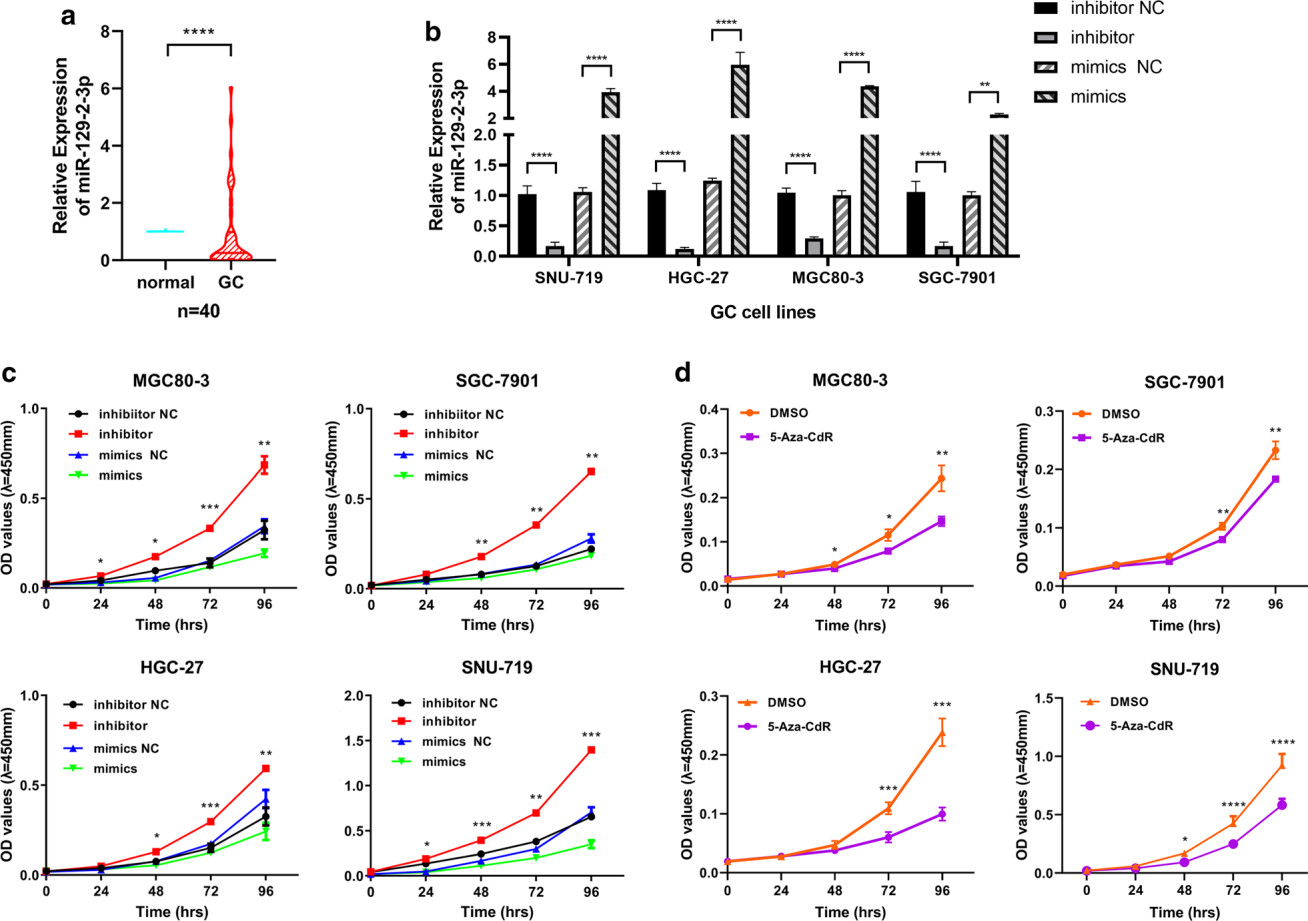

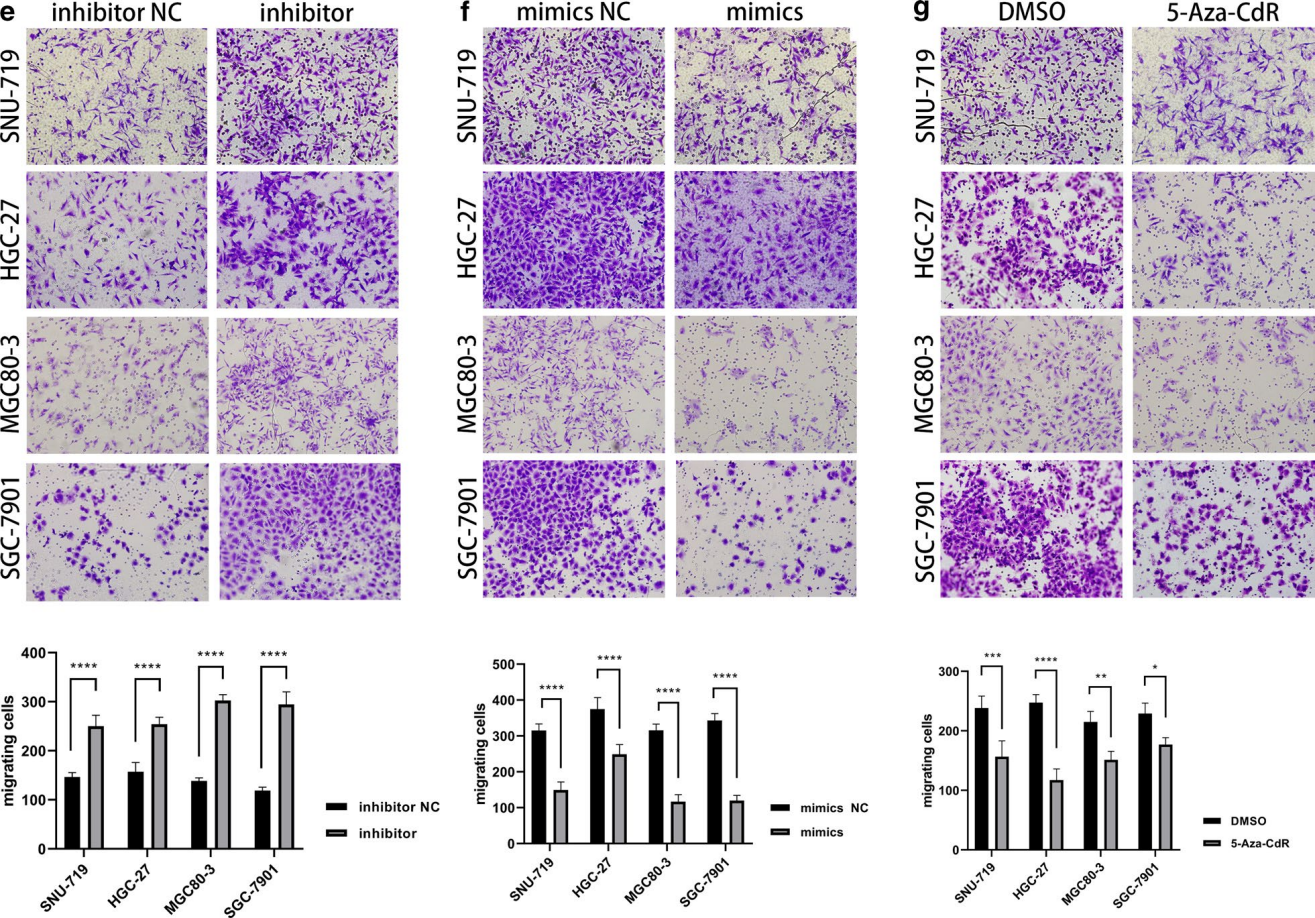

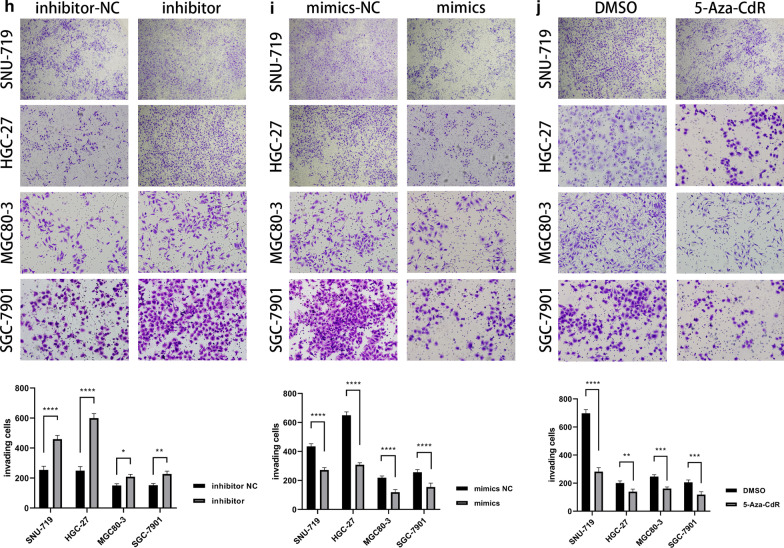
The expression of miR-129-2-3p in GC and the impact on cell functions. **a** The expression of miR-129-2-3p was significantly lower in tumor tissues in 26 cases and higher in 8 cases, while the rest of the cases did not show statistical significance. **b** Transfection efficiency was tested by qPCR. **c** The proliferation capacity of MGC80-3, SGC-7901, HGC-27 and SNU-719 was significantly enhanced after transfection with inhibitor compared to NC while attenuated after transfection with mimics. **d** Comparing to DMSO, the proliferation ability of all these cell lines was sabotaged after treated with the demethylator, 5-Aza-CdR, at 1.5 μmol/L for 3 days. **e**, **f**, **h**, **i** The migration and invasion ability of the GC cells was significantly enhanced after transfected with inhibitor while attenuated after transfection with mimics. **g**, **j** Comparing to DMSO, the migration and invasion capacity of the GC cells was diminished after treatment of 5-Aza-CdR at 1.5 μmol/L for 3 days. *P < 0.05; **P < 0.01; ***P < 0.001; ****P < 0.0001

The genome methylation profiles of TCGA-STAD and TCGA-EBV were displayed in circos plots, respectively (Additional file 2: Fig. S2). We also performed QPCR with the 40 cases of GC samples from our center and discovered that miR-129-2-3p was suppressed in 26 cases of GC and up-regulated in 8 cases, while its expression changes did not reach statistical significance in 6 cases (Fig. 2a). As it turned out, miR-129-2-3p was significantly down-regulated in GC samples (*P* < 0.0001) and might be pivotal for the development for GC. Moreover, according to the sequencing data, the probe for miR-129-2-3p was cg15556502, located at its enhancer region chr11:43602545-43603215. To confirm this result, we have supplemented with a Bisulfite Sequencing PCR (BSP) using the EBVaGC cell line SNU-719 and the EBVnGC cell line MGC80-3, HGC-27 and SGC-7901. As it turned out, there were 2 significantly higher methylated regions in SNU-719 than its EBVnGC counterpart. While the enhancer region was predicted to be rich in methylation, the actual methylated area located at about 500 bp from the transcription start site of miR-129-2-3p, according to the BSP results (Additional file 7: Fig. S19).

### Down-regulation of miR-129-2-3p contributed to enhanced proliferation, motility and invasion of GC cells

To validate the role of miR-129-2-3p in the development of GC, we transfected GC cell lines (SNU-719, MGC80-3, HGC-27, SGC-7901) with miR-129-2-3p mimics, inhibitors and the corresponding negative controls to perform CCK8 and transwell assays. Moreover, we treated all four GC cell lines with 5-Aza-CdR to test the functional changes after demethylation. As could be seen, the efficacy of transfections and 5-Aza-CdR treatment was validated by qPCR (Fig. 2b). After transfection with miR-129-2-3p mimics or treated with 5-Aza-CdR, the proliferative (Fig. 2c, d), migrating (Fig. 2e–g) and invasive behavior (Fig. 2h–j) of GC cells were attenuated, while these capacities were strengthened after miR-129-2-3p were down-regulated by transfection of inhibitor.

### Construction of miRNA-mRNA regulation signaling pathway and validation of DEGs

**Fig. 3:**
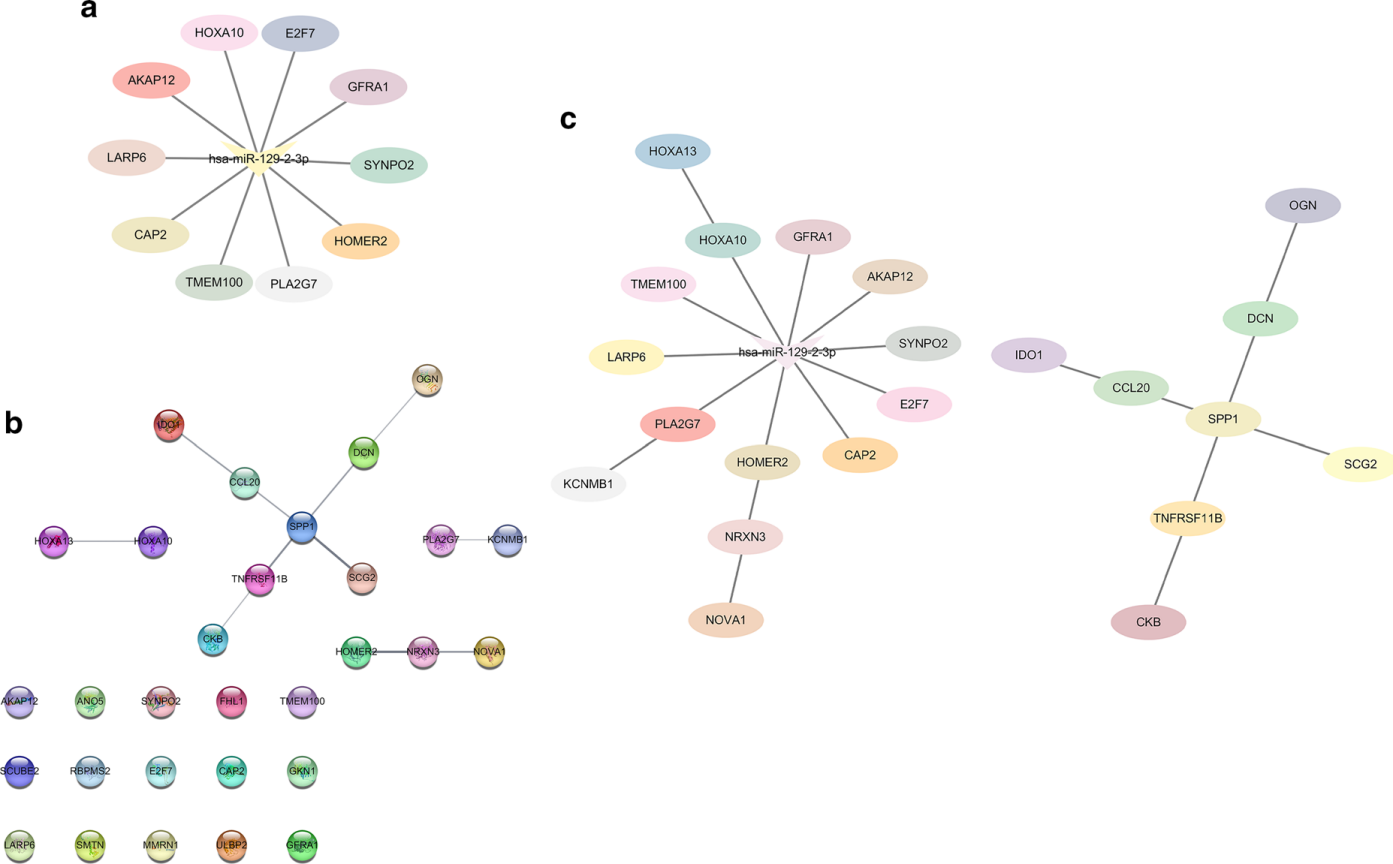

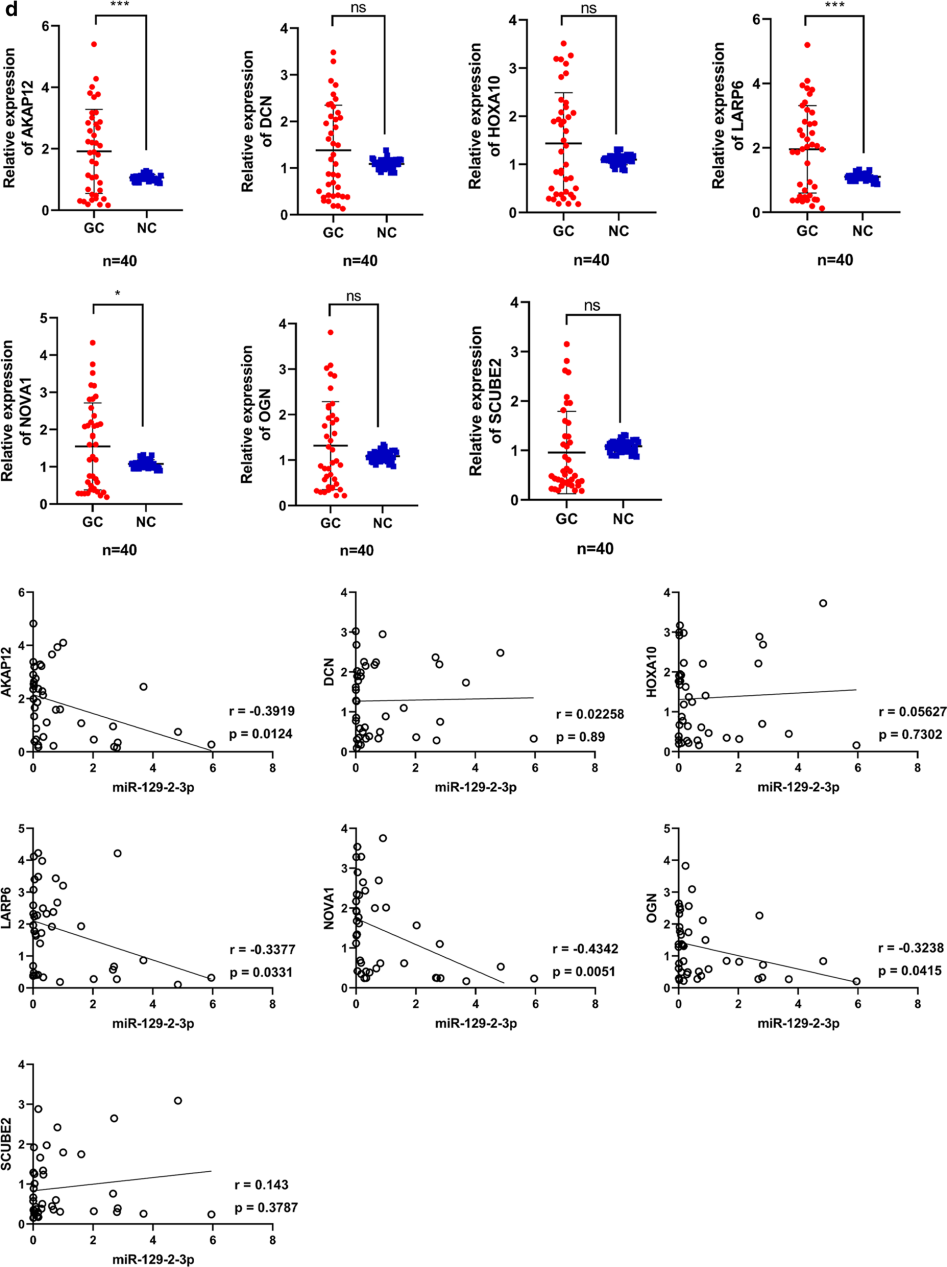

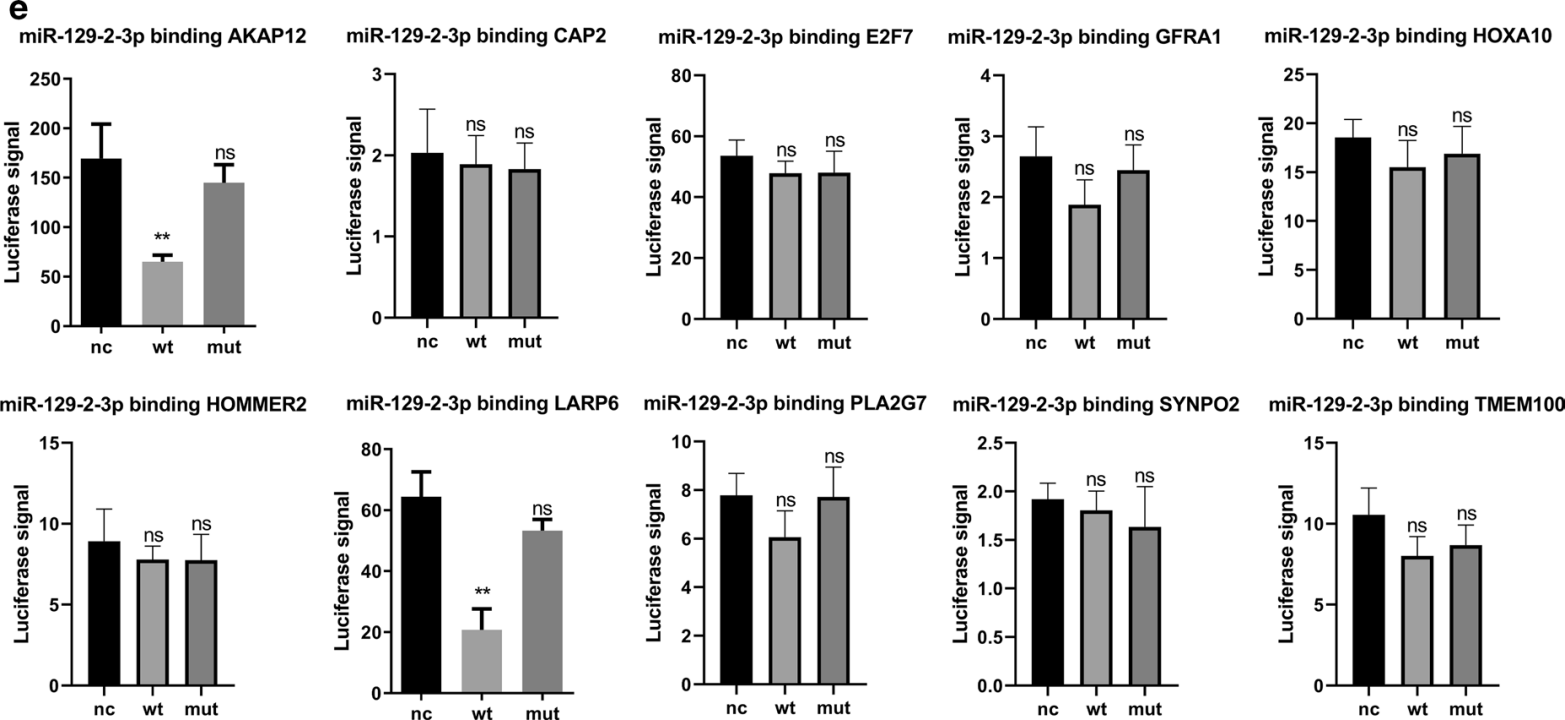
The putative target genes of miR-129-2-3p and the PPI network. Target genes were screened using different online databases (miRMap, miRanda, miRDB, TargetScan and miTarBase). Among the 10 genes, HOMER2 and TMEM100 were validated in 3 different databases (miRDB, miRanda and miRMap), while AKAP12, PLA2G7, HOXA10 and LARP6 were confirmed in both miRanda and miRMap. **a** The miRNA-mRNA regulation network between miR-129-2-3p and its putative target genes was established using a series of online database. **b** PPI network was constructed using STRING database and visualized with Cytoscape. **c** A comprehensive regulation network was established by combining the miRNA-mRNA and PPI networks. **d** The quantification of expression of the putative target genes and their correlations with miR-129-2-3p in GC samples. **e** Dual luciferase reporting system to test the binding activity of the putative target genes and miR-129-2-3p

We used the 5 online databases (seen in Methods and materials) to predict miRNA-mRNA regulation networks. As could be seen, there were 10 genes (LARP6, AKAP12, HOXA10, E2F7, GFRA1, SYNPO2, HOMER2, PLA2G7, TMEM100, CAP2) that might share a 3′-UTR binding site with miR-129-2-3p (Fig. 3a). Among them, HOMER2 and TMEM100 were validated in 3 different databases (miRDB, miRanda and miRMap), while AKAP12, PLA2G7, HOXA10 and LARP6 were confirmed in both miRanda and miRMap. Correlation analysis based on the expression levels of miR-129-2-3p and the putative target genes showed a negative correlation between miR-129-2-3p and AKAP12, LARP6, NOVA1 and OGN (Fig. 3d). To validate the binding effect of miR-129-2-3p and these genes, dual luciferase reporter assays were performed. As could be seen, overexpressing miR-129-2-3p significantly decreased the luciferase activity of wild type AKAP12 and LARP6 and scarcely affect that of their mutant counterparts (Fig. 3e).

As for the analysis of DEGs in GC and normal tissues and EBV, we apply the same cross-matching procedure as the above and found that in the TCGA datasets, there were 1,573 DEGs from the tumor subset and 925 DEGs from the EBV subset, with 421 DEGs in the overlapping area, indicating DEGs of EBVaGC. In the GEO datasets, DEGs from the tumor subset and EBV subset were 107 and 4,276, respectively, with 23 genes as DEGs of EBVaGC (Additional file 1: Fig. S1b). By cross-matching the findings in TCGA and GEO datasets, 30 DEGs were found to be associated with methylation in EBVaGC (Additional file 1: Fig. S1b; Additional file 3).

### PPI network construction

In order to explore the downstream of the miR-129-2-3p regulation network, we analyzed the 30 DEGs with the STRING database for PPI network construction (Additional file 1: Fig. S1b). Hub genes were selected by CytoHubba package in Cytoscape. As a result, 30 nodes and 11 edges were found in the PPI network (Fig. 3b). To establish a comprehensive regulation relationship between miR-129-2-3p and the hub genes, the network added up to 23 nodes and 21 edges, where miR-129-2-3p interacted with the 10 putative targets described above and that HOXA10 was connected with HOXA13, PLA2G7 with KCNMB1 and HOMER2 with NRXN3 and NOVA1 (Fig. 3c).

### Prognostic validation of hub genes

**Fig. 4:**
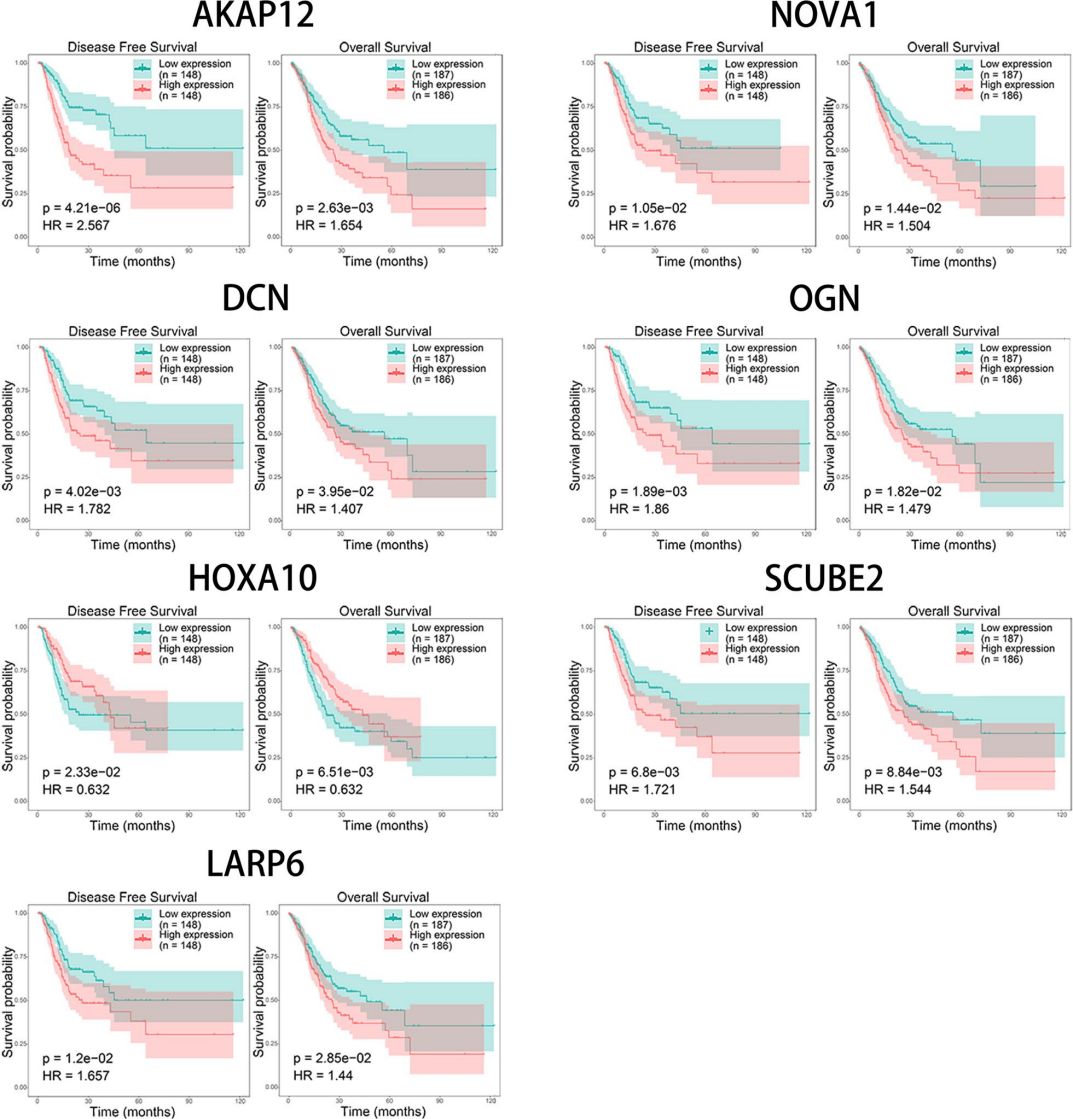
Survival analysis for the hub genes. Among the 30 DEGs, there were 7 genes significant for both disease free survival (left) and overall survival (right). Red curves represented cases with high expression of the corresponding genes and green curves for the cases with low expression. With the exception of HOXA10, all other genes were negatively correlated with patients’ survival

We performed survival analysis for the 30 DEGs selected. Of these, AKAP12, DCN, HOXA10, LARP6, NOVA1, OGN and SCUBE2 were significant for both disease-free survival (DFS) and overall survival (OS). As could be seen, AKAP12, DCN, LARP6, NOVA1, OGN and SCUBE2 were negatively correlated with DFS and OS, while a high level of HOXA10 indicated better survival (Fig. 4). The receiver operating curve (ROC) analysis was also conducted for each of the above DEGs and resulted a series of time-dependent AUCs, which were generally above 0.6, suggesting that these hub genes could assist prognosis evaluations (Additional file 4).

### Transcription factor analysis

**Fig. 5:**
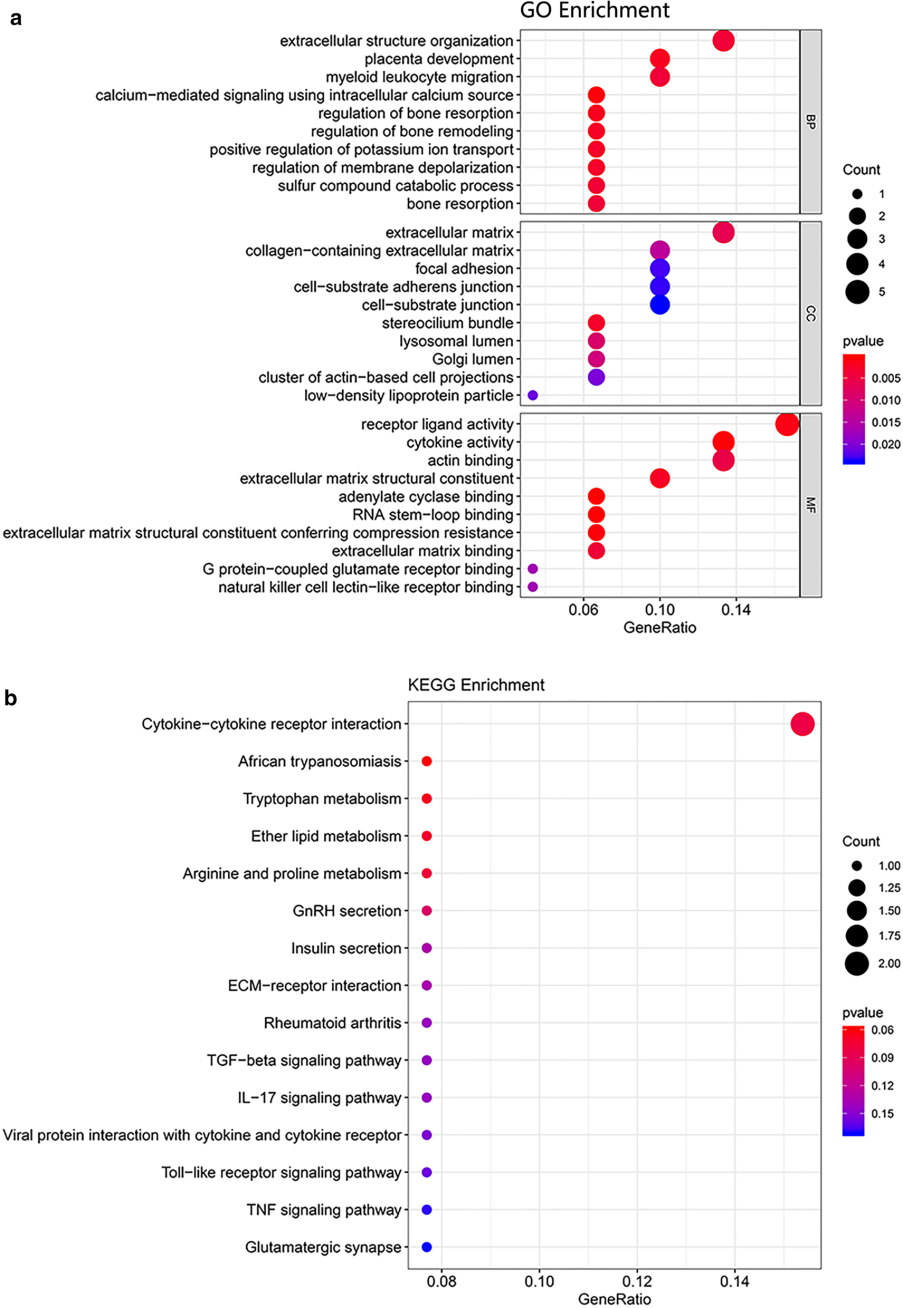
Gene ontology (GO) and Kyoto Encyclopedia of Genes and Genomes (KEGG) enrichment analysis. **a** The biological process analysis revealed extracellular structure organization, myeloid leucocyte migration processes were significantly enriched. In the cell component analysis, extracellular matrix was significantly enriched. In the molecular function analysis, notably, receptor ligand activity, cytokine activity, extracellular matrix binding and other relative processes were significantly enriched. **b** Accordingly, in KEGG analysis, cytokine-cytokine receptor interaction, among others, was significantly enriched

Using iRegulon package in R, we discovered that the most enriched TF binding motifs in the miRNA-mRNA-PPI network were jaspar-CN0215.1 and encode-UW.Motif.0357 and the corresponding TFs were predicted to be STAT3 and NFYC, respectively (Fig. 5a–d). Thus, we were able to postulate a regulating network by integrating miRNA-mRNA-PPI signaling pathways (Fig. 5e), where the transcription of AKAP12, PLA2G7, DCN and OGN was activated by STAT3 and the former 2 genes were post-transcriptionally regulated by miR-129-2-3p. NOVA1, PLA2G7, LARP6, HOXA10, AKAP12 and DCN, on the other hand, were transcriptionally activated by NFYC. Apart from DCN, all of these 5 genes contained 3′-UTR binding site for miR-129-2-3p.

### Function enrichment analysis

**Fig. 6:**
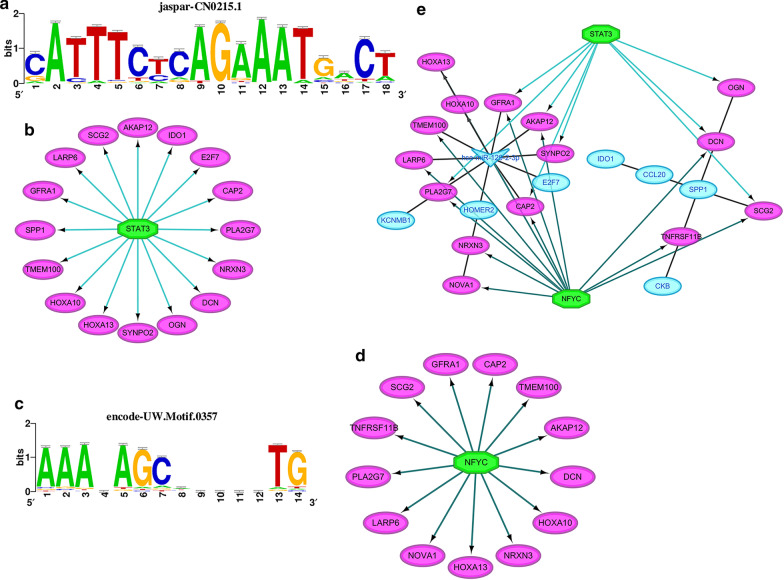
The putative transcription factors and the corresponding binding motifs of the DEGs. **a**, **b** The putative binding motif of STAT3 and its downstream DEGs. **c**, **d** The putative binding motif of NFYC and its regulating DEGs. **e** The comprehensive network integrating the miR-129-2-3p/DEGs and PPI network

To further explore the underlying biological process (BP), molecular function (MF) and cellular component (CC) enrichment of MDEGs in EGVaGC, we used the clusterProfiler package in R for GO analysis. Those with *P* < 0.05, minimum count of 3 and an enrichment factor > 1.5 (which is the ratio of the observed counts and expected counts) were collected and categorized into clusters. The analysis revealed that MDEGs from EBVaGC were particularly enriched in various microenvironment remodeling processes, such as T cell activation, extracellular structure organization, myeloid leucocyte migration, collagen biosynthesis regulation, and so on. Accordingly, the genes enriched for CC and MF were mainly related to extracellular matrix, receptor-ligand activity and cytokine activity, respectively (Fig. 6a). Among these enriched pathways, several DEGs were of peculiar interest. CCL20, for example, could be seen in cytokine activity and myeloid leucocyte migration processes; DCN, on the other hand, which was one of the putative targets of miR-129-2-3p, was enriched in the processes related to extracellular matrix constitution. As for KEGG analysis, meanwhile, cell adhesion molecules, Th17, Th1, Th2 cell differentiation, cytokine-cytokine receptor interaction processes were significantly enriched (Fig. 6b). Collectively, these enrichment analyses suggested that microenvironment remodeling, immune cell activation and differentiation in particular, might exert certain impact on EBVaGC development.

### Immune cell infiltration of EBVaGC

To further explore the EBV-associated microenvironment remodeling effect, we employed the online system, TIMER, to comprehensively analyze the profile of tumor-infiltrating immune cells (TIICs). Specifically speaking, the immune cells for analysis were B cell, CD8^+^ T cell, CD4^+^ T cell, macrophage, neutrophil and dendritic cell. According to the results above, we selected the 7 DEGs that were significantly correlated with survival for analysis and found that AKAP12 and OGN were significantly associated with the 6 types of TIICs during EBVaGC development. DCN and HOXA10 were associated with the 5 types of TIICs, with the exception of B cell. Apart from neutrophil, NOVA1 was also associated with all the other types of TIICs. SCUBE2 and LARP6 were associated with 4 and 3 types of TIICs, respectively (Additional file 5: Fig. S17). Since CD4^+^ T cell, macrophage and dendritic cell were among the TIIC profile of all the 7 DEGs, of which 6 genes were negatively correlated with survival, we thereby speculated that the infiltration of these immune cells might indicate a poor survival in patients of EBVaGC.

## Discussion

Epigenetic modifications are essential for the regulation of gene expression, thereby dynamically affecting physiological as well as pathological processes, especially tumor development. Recent evidence demonstrated that DNA methylation might vary according to the alterations of tumor microenvironment (TME) [23, 24]. Such characteristics could not only be used for the evaluation of the potential of carcinogenesis and metastasis, but also the heterogeneity of TME [24], including the profile of immune cell infiltrations. While the efficacy of immune checkpoint blockade (ICB) therapy against GC has been confirmed by the KEYNOTE-061 study [25], researchers showed that the level of different types of immune cell infiltrations could impose a significant impact on patient overall survival [26]. Therefore, uncovering the immune infiltration profile could facilitate the understanding of how the tumor cells escape the immune system and the prediction of efficacy of ICB. Notably, multiple reports have revealed that EBVaGC has higher ORR in PD-1 inhibitors and better survival than EBVnGC [11, 27], suggesting that the immune cell infiltration profile of EBVaGC might hold great importance. Previously, CD8^+^ T cell was discovered to be a predominant TIIC in EBVaGC [28] and that its abundance was positively correlated with PD-L1 expression [3]. However, cellular immunotherapy harnessing EBV-specific CD8^+^ cytotoxic T cell has merely achieved limited success [29]. Some experts argued that functional systemic CD4 immunity was a prerequisite for efficacious PD-L1/PD-1 blockade therapy [30]. Another assumption for the above dilemma was the counteracting effect of the regulatory T cell (Treg) [31]. Sure enough, subpopulations of CD4^+^ T cells were discovered to hinder effective immune responses against cancer cells [32] as well as to interact with tumor vasculature to promote tumor angiogenesis [33]. In the case of EBVaGC, the profile of TIIC might be correlated with DNA methylation of the host genome [24]. According to our TIIC analysis, CD4^+^ T cell, macrophage and dendritic cell were significantly enriched when the expression of the 6 prognostic-indicated hub genes were elevated. Among the cytokine-activity-related enriched genes in GO and KEGG analysis, CCL20 was the most consistently presented. CCL20 was the ligand of CCR6 and reported to be accountable for recruiting CD4^+^ T cells to promote STAT3 activation to foster cancer stemness [34]. We therefore hypothesized that the hypermethylation events in EBVaGC triggered secretion of cytokines like CCL20 to create an immunosuppressive microenvironment, which contained the aforementioned TIIC profile.

Apart from the distinct immune infiltration profile related to DNA methylation, the abundance of methylation itself was reported to be correlated with disease progression [35]. Due to the strong pro-carcinogenic effect and the lack of eradication therapy for EBV [36], targeting methylation might be a useful strategy for prevention of EBVaGC [15, 37]. Unfortunately, however, most of the methylation-targeted regimens under clinical trials were designated for genome-wide utilization [38]. Side effects are inevitable. In order to be more precise in treatment, we have to explore the mechanism of DNA methylations in EBVaGC and the potential target sites for demethylation.

The underlying mechanism for DNA methylation and its biological impacts on cancer cells are beginning to come into light. Currently, researchers discovered that EBV could directly modulate the expression of DNMT1 and TET2 demethylase by expressing latent genes, such as LMP1 and EBNA1, to arouse hypermethylation of the host genome [13, 14, 39], during which certain genes could be methylated in a de novo pattern [39]. Notably, the aberrant methylation mainly composed of promoter/CpG island hypermethylation of multiple tumor suppressor genes [40], activating signaling pathways like NF-κB, phosphoinositide 3-kinase/Akt pathways [23] and pathways related to focal adhesion formation, cytokines and receptors interactions, actin cytoskeleton regulation, and so on [40]. In accordance with the previous studies, after analyzing the aforementioned RNA-seq and mRNA profiling data from the TCGA-STAD and GEO database, we found that pathways regulating receptor-ligand activity, cytokine activity, actin binding, extracellular structure organization were significantly enriched in GO analysis, while cytokine-cytokine receptor interaction was significantly enriched in KEGG analysis. In the perspective of therapeutic targeting, however, treatment against EBVaGC still remains a challenge since these signaling pathways are not specific enough.

As an important composition of post-transcriptional regulation, miRNAs target up to 60% of coding genes in human [41]. The expression level of certain miRNAs was reported to be lineage specific [18, 19], enabling precise targeting. In the case of EBVaGC, miRNAs could originate from both the genomes of EBV and the host and could be modified by DNA methylation like other encoding genes [42]. But the role of methylation of miRNA in the progression of EBVaGC is still obscure.. In this study, we found that miR-129-2-3p was the only one miRNA that reached statistical significance in both STAD/normal and methylation profiling datasets. Notably, the hypermethylation was predicted to occur in the enhancer region 200 bp upstream of the transcription starting site (TSS). Recently, a research group has demonstrated that DNA methylation at enhancers was correlated with distinct cancer lineages [16]. This indicated that the enhancer methylation of miR-129-2-3p might be a specific marker for identification of EBVaGC.

Previously, chromosomal aberration investigations showed that loss of 11p was an exclusive characteristic restricted to EBVaGC [43]. miR-129-2-3p is a cancer-associated miRNA located at 11p11.2. It acted as a tumor suppressor gene by inducing cell cycle arrest [44] and its level in gastric juice could be utilized as a biomarker for screening GC [45]. In order to elucidate the role of miR-129-2-3p, we further validate its expression profile in tissue samples and conducted cellular function assays. Indeed, in our findings, miR-129-2-3p was significantly down-regulated in GC samples comparing to the adjacent normal tissues detected by QPCR. The proliferative, migrative and invasive capacities of GC Cells were suppressed when transfected with miR-129-2-3p mimics and enhanced with inhibitors. Using online tools to predict miRNA-mRNA interactions, we discovered that HOMER2, TMEM100, AKAP12, PLA2G7, HOXA10 and LARP6 were potential targets of miR-129-2-3p, consistent at least in 2 databases. Prognostic validation of these genes was also performed. AKAP12, HOXA10 and LARP6 were negatively correlated with DFS and OS.

Moreover, with the help of iRegulon package, we were able to predict TFs and the corresponding binding motifs in the promoter region of the prognostic-indicated hub genes. As was demonstrated, STAT3 and NFYC were the most likely TFs among others to bind to the motifs of jaspar-CN0215.1 and encode-UW.Motif.0357, respectively. Among the hub genes described above, STAT3 served as the transcription activator for AKAP12, PLA2G7, DCN and OGN, while NFYC was for NOVA1, PLA2G7, LARP6, HOXA10, AKAP12 and DCN. To clarify the regulation network based on miR-129-2-3p, we integrated the miRNA-mRNA regulation network and the PPI analysis. Thus came the interaction network demonstrated above.

In conclusion, we have utilized public databases and in vitro experiments to analyze the role of DNA methylation in the development of EBVaGC, including the establishment of miRNA-mRNA-PPI regulation network and association of immune cell infiltration. From the results, we were able to deduce the hypermethylation of enhancer of miR-129-2-3p elevated the expression of some prognostic-indicated hub genes, such as AKAP12, HOXA10 and LARP6, and brought about the enrichment of cytokine activity signaling pathways to recruit immune cells like CD4^+^ T cell, macrophage and dendritic cell. Although our investigation was quite comprehensive, we still need to further confirm our results with more extensive in vitro and in vivo assays in the future.

## Conclusions

This study has harnessed multi-source data to present a comprehensive analysis of differentially expressed miRNAs and mRNAs associated with DNA methylation in EBVaGC and demonstrated the oncogenic role of methylation-related suppression of miR-129-2-3p in the development of EBVaGC. By integrating the transcription factor analysis and PPI analysis, we were able to establish a regulation network based on miR-129-2-3p. Moreover, according to the pathway enrichment analysis and TIIC enrichment analysis, we hypothesized that the down-regulation of miR-129-2-3p might be essentially associated with the enriched biological process of cytokine activity and CD4^+^ T cell, macrophage and dendritic cell infiltration. Our findings provided a clue of the role of epigenetic changes of tumor cells in the development in EBVaGC and its possible relationship with immune cell infiltration. Further experimental and clinical investigations are warranted to explore the underlying mechanism and validate our findings.

## Materials and methods

### Clinical samples, RNA extraction and RT-qPCR of miR-129-2-3p

A total of 40 cases of GC were randomly selected from our tissue bank. Each case of GC contained a pair of tumor tissues and adjacent normal tissues. Written informed consent were obtained from patients before surgery at our hospital from 2016 to 2018. Fresh sample tissues were immediately frozen after resection and stored at − 80 ℃. Diagnosis of the GC cases in our hospital was confirmed by 3 independent pathologists using Hematoxylin–Eosin staining of paraffin-embedded sections. Total RNA was extracted from the tissue samples with RNA-Quick Purification Kit (YiShan Biotechnology CO., LTD, Shanghai, China) according to the manufacturer’s protocol. miR-129-2-3p was quantified by real-time PCR. The primers for RT-qPCR were listed in Additional file 6: Table. S1. Briefly, mature miRNA was reversely transcribed from 500 ng of RNA using PrimeScript RT reagent Kit (TaKaRa, Japan). QPCR was carried out using TB Green Premix Ex Taq (TaKaRa, Japan) with ABI QuantStudio 5 (Thermo Fisher Scientific, USA). Small RNA U6 was used as endogenous control for input normalization. The relative miRNA expression was calculated as 2^−ΔΔCT^.

### Gastric cancer cell lines and culture condition

GC cell lines SNU-719, MGC80-3, HGC-27 and SGC-7901 were cultured in RPMI-1640 medium (Gibco, Grand Island, NY), supplemented with 10% fetal bovine serum (Gibco) in a humidified atmosphere containing 5% CO_2_ at 37 ℃. The EBVaGC cell line SNU-719 was provided by Prof. Chunkui Shao from The 3^rd^ Affiliated Hospital of Sun Yatsen University. 5-aza-2′-deoxycytidine (5-Aza-CdR; Sigma-Aldrich, USA) was used for demethylation treatment at 1.5 μmol/L for 3 days as previously indicated [46].

### Oligonucleotide transfection

Lipofectamine 2000 reagent (Invitrogen, Life Technologies, USA) was used to deliver single-stranded RNA molecules (GeneChem, Shanghai, China) into GC cells according to manufacturer’s instruction. Briefly, cells were seeded at 2 × 10^5^ cells per well in a 6-well plate and transfected with 50 nM of mimics or inhibitor of has-miR-129-2-3p (mature sequence: 5′-AAGCCCUUACCCCAAAAAGCAU-3′, miRBase access number: MIMAT0004605) when confluence reached 70%. Equal amount of random sequence miRNA mimics or inhibitor were used as negative controls. Cells were cultured as before and harvested 48 h after transfection for functional assays. Transfection efficiency was evaluated by RT-qPCR (Additional file 6: Fig. S18).

### Cell proliferation assay with cell counting kit-8 (CCK-8)

200 μl of GC cells suspension was seeded into the 96-well plate at 2000 cells per well and incubated for different time periods [0, 24, 48, 72, 96h]. At each time point, 20 μl of CCK-8 (Dojindo, Japan) was added to each well for further incubation for 2 h. The absorbance value of the plate was detected using a microplate analyzer at 450 nm.

### Cell migration and invasion assay

For migration assays, 2 × 10^4^ GC cells in serum-free medium were seeded onto the upper chamber of a Transwell insert with 8 μm pore size polycarbonate membrane (Corning, USA). For invasion assays, the Transwell insert was coated with 20 μg Matrigel (Corning, USA) and seeded with 5 × 10^4^ GC cells on top. The medium containing 10% fetal bovine serum was added in the lower chamber. 24 h later [48hforinvasionassays], a cotton swab was used to remove the cells that remained on the upper layer of the membrane. Then the membrane was fixed with paraformaldehyde and stained with 0.1% crystal violet. 5 different fields from the membrane were randomly selected and the number of migrating or invading cells was counted using an optical microscope (Olympus BX51, Japan).

### Microarray datasets

In the present study, data were collected from GEO (http://www.ncbi.nlm.nih.gov/geo/) and TCGA (http://portal.gdc.cancer.gov/) databases. Since these data are publicly available, approval from the local ethics committee is not required.

For detection of differentially expressed miRNAs (DEmiRNAs) between cancerous and adjacent normal tissues in GC, TCGA-STAD miRNA-seq data and GEO dataset GSE87785 miRNA microarray profiling data were used. For mRNA analysis, TCGA-STAD RNA-seq data and GSE66229 were utilized. To determine the methylated differentially expressed genes (MDEGs) between cancerous and adjacent normal tissues in EBV positive/negative cases, TCGA-STAD 450 k dataset was analyzed. We also employed TCGA-STAD RNA-seq and GSE51575, as well as TCGA-STAD miRNA-seq data to identify the differentially expressed genes (DEGs) and DEmiRNAs between EBV positive and negative cases, respectively.

Within TCGA-STAD miRNA-seq high-throughput sequencing data, there were 41 cases of adjacent normal tissues as control and 436 cases of GC as experimental group (STAD). By application of the GPL11154 platform (Illumina Hiseq 2000), GSE87785 contained 2 samples of normal and STAD, respectively. For mRNA dataset GSE66229, using GPL570 platform (HG-U133_plus_2, Affymetrix Human Genome U133 Plus 2.0 Array), there were 100 normal controls and 300 STAD samples, while there were 32 normal controls and 375 STAD samples in the TCGA-STAD RNA-seq dataset.

In the TCGA-STAD methylation profiling dataset (Illumina HumanMethylation450K), 2 cases of adjacent normal tissues served as control and 395 cases of GC specimens were categorized as STAD group.

For EBV associated DEGs identification, TCGA-STAD RNA-seq dataset contained 217 cases of negative specimens and 23 positive tissue samples. With the platform of GPL13607 (Agilent-028004 SurePrint G3 Human GE 8 × 60 K Microarray), GSE51575 dataset contained 14 EBV negative cases and 12 positive ones. During the identification EBV associated DEmiRNAs, 269 cases of EBV negative samples and 26 cases of positive samples were selected after excluding the ones without information on EBV infection in TCGA-STAD miRNA-seq dataset.

### Gene expression and methylation analysis

Data of gene expression and methylation from TCGA-STAD and GEO datasets were analyzed with R 3.5.1 software (http://www.r-project.org/) after normalization. mRNA and miRNA sequencing data were analyzed with edgeR package, while mRNA profiling microarray was analyzed with limma package. Although the dataset GSE87785 offered sequencing data, it was shown as an RPKM matrix. Thus, we processed that dataset with limma package as well. Cutoff criteria were set as adjusted *P* value < 0.05 and fold change (FC) > 2 or < 1/2 (|log_2_FC|> 1) for mRNA sequencing data and FC > 1.5 or < 1/1.5 (|log_2_FC|> 0.585) for miRNA sequencing and mRNA microarray profiling. Moreover, ChAMP package was used for analyzing differentially methylated CpG sites (DMCs) and differentially methylated genes (MDEGs) in microarray datasets. All DMCs were annotated into the corresponding MDEGs based on the platform annotation file. After downloading β values, the data underwent quality control, screening and normalization. In the screening process, multi-hit probes and probes on the X and Y chromosomes were excluded. The online software GEO2R (http://www.ncbi.nlm.nih.gov/geo/geo2r/) was utilized to analyze the raw data to identify DEGs. *P* < 0.05 and |FC|> 2 were considered as cutoff criteria of DEGs. To strengthen the credibility of our results, we integrated TCGA-STAD RNA-seq and GSE66229 to render cross-matched genes for DEGs between STAD and normal samples, TCGA-STAD RNA-seq and GSE51575 for DEGs between EBV positive and negative samples, and TCGA-STAD miRNA-seq and GSE87785 for DEmiRNAs between STAD and normal samples. Finally, the cross-matched genes from EBV cohorts and GC cohorts were further integrated with MDEGs to yield methylation associated DEGs/DEmiRNAs in EBVaGCs, which was presented by Venn diagram.

### miRNA-mRNA regulation network construction

After methylation associated DEmiRNAs were detected, 5 databases (miRMap, miRanda, miRDB, TargetScan and miTarBase) were used to establish the interaction between miRNAs and mRNAs. The miRNA-mRNA interactions that were validated by at least 2 databases were selected and visualized by Cytoscape (http://cytoscape.org/).

### Protein–protein interaction (PPI) network analysis

To further clarify the interactive relationships among MDEGs, PPI analysis was performed using Retrieval of Interacting Genes (STRING) database (http://string-db.org/). PPIs with a combined score > 0.4 were reserved for further analysis. Cytoscape was put into use to display the selected PPI networks and CytoHubba was utilized to identify the top hub genes, which were further selected and ranked by integrating 12 topological methods, including Maximal Clique Centrality, Degree, Edge Percolated Component, Maximum Neighborhood Component, Density of Maximum Neighborhood Component, Maximal Clique Centrality, and six centralities (Bottleneck, EcCentricity, Closeness, Radiality, Betweenness, and Stress) CytoHubba provided.

### Gene ontology and pathway enrichment

Gene Ontology Biological Process enrichment analysis was performed with the online tool Metascape (http://metascape.org) to annotate different biological processes of MDEGs. Genes were clustered based on their pathways and the statistical significance of genes was determined upon the enrichment score in each biological process. Subsets of enriched terms were selected and rendered as network plots, in which those with a similarity > 0.3 were connected by edges, and visualized by Cytoscape. The signaling pathways underlying EBVaGC development for the selected MDEGs were depicted by Kyoto Encyclopedia of Genes and Genomes (KEGG) pathway enrichment analysis using the R package clusterProfiler. The correlations between MDEGs and enriched pathways were determined using Fisher exact test. *P* < 0.05 or adjusted *P* < 0.1 was regarded as statistically significant.

### Prognosis related hub genes screening

Clinical data from TCGA-STAD were used to screen to prognosis related hub genes.

### Immune cell infiltration analysis

To determine the correlations between the MDEGs and abundance of the 6 types of tumor infiltrated immune cells (B cells, CD8^+^ T cells, CD4^+^ T cells, macrophage, neutrophil, dendritic cells), the web server, TIMER [47] (http://cistrome.shinyapps.io/timer/), was put into use.

### Transcription factor analysis

To identify the potential TF regulating the DEGs, a Cytoscape plugin, iRegulon [48], was used.

### Statistical analysis

Statistical analyses were performed using IBM SPSS version 24.0 and GraphPad Prism 5.0 (GraphPad Software Inc., CA, USA). Association with clinicopathological variables was examined using Mann–Whitney test. Survival analyses were performed with Kaplan–Meier curves and log-rank test. The cell functional assays were evaluated using the one-way or two-way ANOVA. QPCR array were measured with Mann–Whitney *U* test. A two-tailed *P* < 0.05 was considered statistically significant. All the statistics were expressed as mean ± standard deviation (SD) of three independent experiments.

## Supplementary Information

**Additional file 1: Figure S1.** Venn Diagram showing the details of crossmatch of the multiple datasets. The display and analysis of genome methylation profile of GC and EBV cases. (a) Differentially expressed miRNAs of GC were rendered after the crossmatch of TCGA miRNA-seq and GSE87785, while differentially expressed methylated miRNAs were discovered using TCGA-methylation GC dataset. The EBV-related differentially expressed methylated miRNAs were analyzed using TCGA sequencing data. There was only one miRNA, miR-129-2-3p, that was simultaneously fit the GC and EBV criteria. (b) Differentially expressed genes (DEGs) were determined after crossmatching the GC datasets of TCGA RNA-seq and GSE66229 and the EBV datasets of TCGA RNA-seq and GSE51575. 30 DEGs were finally discovered.

**Additional file 2: Figure S2.** Circos plot depicting the genome-wide methylation profile of GC (a) and EBV (b) cases from the TCGA database. Hypermethylation is represented by red colors and hypomethylation by blue. From the inner circles to outer layer are hypomethylation peak plot, hypermethylation peak plot and methylation scatter plot.

**Additional file 3:** Differentially expressed genes due to methylation in EBVaGC.

**Additional file 4: Figure S3.** The time-dependent ROC curves for AKAP12, with the highest AUC of 0.612 at 60 and 65 months. **Figure S4.** Multivariate (up) and univariate (down) Cox-regression for AKAP12. **Figure S5.** The time-dependent ROC curves for DCN, with the highest AUC of 0.623 at 60 and 65 months. **Figure S6.** Multivariate (up) and univariate Cox-regression of DCN. **Figure S7.** The time-dependent ROC curves for LARP6, with the highest AUC of 0.601 from 75 months to 120 months. **Figure S8.** Multivariate (up) and univariate (down) Cox-regression of LARP6. **Figure S9.** The time-dependent ROC curves for NOVA1, with the highest AUC of 0.565 at 50 and 55 months. **Figure S10.** Multivariate (up) and univariate (down) Cox-regression of NOVA1. **Figure S11.** The time-dependent ROC curves for OGN, with the highest AUC of 0.609 at 5 months. **Figure S12.** Multivariate (up) and univariate Cox-regression of OGN. **Figure S13.** The time-dependent ROC curves of SCUBE2, with the highest AUC of 0.710 at 70 months. **Figure S14.** Multivariate (up) and univariate (down) Cox-regression of SCUBE2. **Figure S15.** The time-dependent ROC curves of HOXA10, with the highest AUC of 0.606 at 5 months. **Figure S16.** Multivariate (up) and univariate (down) Cox-regression of HOXA10.

**Additional file 5: Figure S17.** Tumor infiltrating immune cell analysis of the 7 hub genes. (a, b) AKAP12 and OGN were correlated with the enrichment of all the 6 types of TIICs, with particularly higher partial correlation coefficients in CD4+ T cell and macrophage. (c, d) DCN and HOXA10 were correlated with the enrichment of all the TIICs except B cell, with a particularly higher partial correlation coefficient in macrophage. (e) NOVA1 was correlated with all types of TIICs except neutrophil, with particularly higher partial correlation coefficients in CD4+ T cell and macrophage. (f) SCUBE2 was associated with the enrichment of B cell, CD4+ T cell, macrophage and dendritic cell, also with higher partial correlation coefficients in CD4+ T cell and macrophage. (g) LARP6 was associated with the enrichment of CD4+ T cell, macrophage and dendritic cell, with the highest partial correlation coefficient in macrophage.

**Additional file 6: Table S1.** Primers for QPCR assays. **Figure S18.** The relative expression of miR-129-2-3p of different GC cell lines transfected with inhibitor/mimics. **P=0.0013, ****P<0.0001.

**Additional file 7: Figure S19.** Bisulfite Sequencing PCR (BSP) to test the methylation level of the EBVaGC cell line SNU-719 and the EBVnGC cell lines MGC80-3, HGC-27 and SGC-7901. Group1, MGC80-3, HGC-27 and SGC-7901; Group 2, SNU-719. (a) There are 4 regions rich in methylation, 404-510bp, 1026-1211bp, 1700-1914bp and 1949-2054bp from the TSS of miR-129-2-3p. (b) Regions 1 and 4 in SNU-719 are significantly higher methylated than its EBVnGC counterpart.

**Additional file 8: Figure S20.** In situ hybridization (ISH) assay using EBER probe to detect EBV infection in GC cells.

**Additional file 9: Figure S21.** The endogenous expression of miR-129-2-3p in different GC cell lines.

**Additional file 10: Figure S22.** Expression changes in miR-129-2-3p in the GC cell lines by the treatment of demethylator 5-Aza-CdR.

**Additional file 11: Figure S23.** Dual Luciferase Reporting system to validate the binding activity of miR-129-2-3p to the 3’-UTR of target genes.

**Additional file 12: Figure S24.** The expression changes of the putative target genes transfected with mimics or inhibitor of miR-129-2-3p.

**Additional file 13: Figure S25.** Survival analysis of the target genes combining miR-129-2-3p.

**Additional file 14: Figure S26.** The expression changes of the putative target genes transfected with siRNAs of STAT3.

## Data Availability

The microarray and high-throughput sequencing data of miRNA and mRNA as well as the methylome datasets were retrieved from the TCGA and GEO databases.

## Abbreviations

EBV: Epstein–Barr virus
GC: Gastric cancer
EBVaGC: EBV-associated gastric cancer
DFS: Disease free survival
OS: Overall survival
TCGA: The Cancer Genome Atlas Research Network
GEO: The Gene Expression Omnibus
DEG: Differentially expressed gene
DMC: Differentially methylated CpG site
MDEG: Methylated differentially expressed gene
PPI: Protein–protein interaction
TIIC: Tumor infiltrating immune cell
QPCR: Quantitative polymerase chain reaction
CCK8: Cell counting kit 8
ROC: Receiver operating characteristic curve
AUC: Area under curve

## References

[1] Chen W, Zheng R, Baade PD, Zhang S, Zeng H, Bray F (2016). Cancer statistics in China, 2015. CA Cancer J Clin.

[2] Cancer Genome Atlas Research N (2014). Comprehensive molecular characterization of gastric adenocarcinoma. Nature.

[3] Thompson ED, Zahurak M, Murphy A, Cornish T, Cuka N, Abdelfatah E (2017). Patterns of PD-L1 expression and CD8 T cell infiltration in gastric adenocarcinomas and associated immune stroma. Gut.

[4] Yoda Y, Takeshima H, Niwa T, Kim JG, Ando T, Kushima R (2015). Integrated analysis of cancer-related pathways affected by genetic and epigenetic alterations in gastric cancer. Gastric Cancer.

[5] Asada K, Nakajima T, Shimazu T, Yamamichi N, Maekita T, Yokoi C (2015). Demonstration of the usefulness of epigenetic cancer risk prediction by a multicentre prospective cohort study. Gut.

[6] Kupcinskaite-Noreikiene R, Ugenskiene R, Noreika A, Rudzianskas V, Gedminaite J, Skieceviciene J (2016). Gene methylation profile of gastric cancerous tissue according to tumor site in the stomach. BMC Cancer.

[7] Oue N, Oshimo Y, Nakayama H, Ito R, Yoshida K, Matsusaki K (2003). DNA methylation of multiple genes in gastric carcinoma: association with histological type and CpG island methylator phenotype. Cancer Sci.

[8] Kurklu B, Whitehead RH, Ong EK, Minamoto T, Fox JG, Mann JR (2015). Lineage-specific RUNX3 hypomethylation marks the preneoplastic immune component of gastric cancer. Oncogene.

[9] Fang WL, Chen MH, Huang KH, Chang SC, Lin CH, Chao Y (2019). Analysis of the clinical significance of DNA methylation in gastric cancer based on a genome-wide high-resolution array. Clin Epigenetics.

[10] Matsusaka K, Kaneda A, Nagae G, Ushiku T, Kikuchi Y, Hino R (2011). Classification of Epstein-Barr virus-positive gastric cancers by definition of DNA methylation epigenotypes. Cancer Res..

[11] Namba-Fukuyo H, Funata S, Matsusaka K, Fukuyo M, Rahmutulla B, Mano Y (2016). TET2 functions as a resistance factor against DNA methylation acquisition during Epstein-Barr virus infection. Oncotarget..

[12] Ma G, Liu H, Hua Q, Wang M, Du M, Lin Y (2017). KCNMA1 cooperating with PTK2 is a novel tumor suppressor in gastric cancer and is associated with disease outcome. Mol Cancer..

[13] Dimitrova N, Gocheva V, Bhutkar A, Resnick R, Jong RM, Miller KM (2016). Stromal expression of miR-143/145 promotes neoangiogenesis in lung cancer development. Cancer Discov..

[14] Kim P, Park A, Han G, Sun H, Jia P, Zhao Z (2018). TissGDB: tissue-specific gene database in cancer. Nucleic Acids Res..

[15] Hattori N, Ushijima T (2016). Epigenetic impact of infection on carcinogenesis: mechanisms and applications. Genome Med..

[16] Anderson BW, Suh YS, Choi B, Lee HJ, Yab TC, Taylor WR (2018). Detection of gastric cancer with novel methylated dna markers: discovery, tissue validation, and pilot testing in plasma. Clin Cancer Res..

[17] Huang KK, Ramnarayanan K, Zhu F, Srivastava S, Xu C, Tan A (2018). Genomic and epigenomic profiling of high-risk intestinal metaplasia reveals molecular determinants of progression to gastric cancer. Cancer Cell..

[18] Farrell PJ (2019). Epstein-Barr virus and cancer. Annu Rev Pathol..

[19] Wang K, Liang Q, Li X, Tsoi H, Zhang J, Wang H (2016). MDGA2 is a novel tumour suppressor cooperating with DMAP1 in gastric cancer and is associated with disease outcome. Gut..

[20] Abdelfatah E, Kerner Z, Nanda N, Ahuja N (2016). Epigenetic therapy in gastrointestinal cancer: the right combination. Therap Adv Gastroenterol..

[21] Kaneda A, Matsusaka K, Aburatani H, Fukayama M (2012). Epstein-Barr virus infection as an epigenetic driver of tumorigenesis. Cancer Res..

[22] Liang Q, Yao X, Tang S, Zhang J, Yau TO, Li X (2014). Integrative identification of Epstein-Barr virus-associated mutations and epigenetic alterations in gastric cancer. Gastroenterology..

[23] Fukayama M, Hino R, Uozaki H (2008). Epstein-Barr virus and gastric carcinoma: virus-host interactions leading to carcinoma. Cancer Sci..

[24] Croce CM (2009). Causes and consequences of microRNA dysregulation in cancer. Nat Rev Genet..

[25] Fleischer T, Tekpli X, Mathelier A, Wang S, Nebdal D, Dhakal HP (2017). DNA methylation at enhancers identifies distinct breast cancer lineages. Nat Commun..

[26] Zur HA, van Grieken NC, Meijer GA, Hermsen MA, Bloemena E, Meuwissen SG (2001). Distinct chromosomal aberrations in Epstein-Barr virus-carrying gastric carcinomas tested by comparative genomic hybridization. Gastroenterology..

[27] Kim ST, Cristescu R, Bass AJ, Kim KM, Odegaard JI, Kim K (2018). Comprehensive molecular characterization of clinical responses to PD-1 inhibition in metastatic gastric cancer. Nat Med..

[28] van Beek J, Zur Hausen A, Snel SN, Berkhof J, Kranenbarg EK, Van de Velde CJ (2006). Morphological evidence of an activated cytotoxic T-cell infiltrate in EBV-positive gastric carcinoma preventing lymph node metastases. Am J Surg Pathol..

[29] Zhang NN, Chen JN, Xiao L, Tang F, Zhang ZG, Zhang YW (2015). Accumulation mechanisms of CD4(+)CD25(+)FOXP3(+) regulatory T cells in EBVassociated gastric carcinoma. Sci Rep..

[30] Zuazo M, Arasanz H, Fernandez-Hinojal G, Garcia-Granda MJ, Gato M, Bocanegra A (2019). Functional systemic CD4 immunity is required for clinical responses to PD-L1/PD-1 blockade therapy. EMBO Mol Med..

[31] Li J, Qian CN, Zeng YX (2009). Regulatory T cells and EBV associated malignancies. Int Immunopharmacol..

[32] Saito T, Nishikawa H, Wada H, Nagano Y, Sugiyama D, Atarashi K (2016). Two FOXP3(+)CD4(+) T cell subpopulations distinctly control the prognosis of colorectal cancers. Nat Med..

[33] Johansson-Percival A, He B, Ganss R (2018). Immunomodulation of tumor vessels: it takes two to tango. Trends Immunol..

[34] Kryczek I, Lin Y, Nagarsheth N, Peng D, Zhao L, Zhao E (2014). IL-22(+) CD4(+) T cells promote colorectal cancer stemness via STAT3 transcription factor activation and induction of the methyltransferase DOT1L. Immunity..

[35] Huang KK, Ramnarayanan K, Zhu F, Srivastava S, Xu C, Tan ALK (2018). Genomic and epigenomic profiling of high-risk intestinal metaplasia reveals molecular determinants of progression to gastric cancer. Cancer Cell..

[36] Farrell PJ (2019). Epstein-Barr virus and cancer. Annu Rev Pathol..

[37] Wang K, Liang Q, Li X, Tsoi H, Zhang J, Wang H (2016). MDGA2 is a novel tumour suppressor cooperating with DMAP1 in gastric cancer and is associated with disease outcome. Gut..

[38] Abdelfatah E, Kerner Z, Nanda N, Ahuja N (2016). Epigenetic therapy in gastrointestinal cancer: the right combination. Therap Adv Gastroenterol..

[39] Kaneda A, Matsusaka K, Aburatani H, Fukayama M (2012). Epstein-Barr virus infection as an epigenetic driver of tumorigenesis. Cancer Res..

[40] Liang Q, Yao X, Tang S, Zhang J, Yau TO, Li X (2014). Integrative identification of Epstein-Barr virus-associated mutations and epigenetic alterations in gastric cancer. Gastroenterology..

[41] Croce CM (2009). Causes and consequences of microRNA dysregulation in cancer. Nat Rev Genet..

[42] Kang D, Skalsky RL, Cullen BR (2015). EBV BART microRNAs target multiple proapoptotic cellular genes to promote epithelial cell survival. PLoS Pathog..

[43] Zur Hausen A, van Grieken NC, Meijer GA, Hermsen MA, Bloemena E, Meuwissen SG (2001). Distinct chromosomal aberrations in Epstein-Barr virus-carrying gastric carcinomas tested by comparative genomic hybridization. Gastroenterology..

[44] Yu X, Song H, Xia T, Han S, Xiao B, Luo L (2013). Growth inhibitory effects of three miR-129 family members on gastric cancer. Gene..

[45] Yu X, Luo L, Wu Y, Yu X, Liu Y, Yu X (2013). Gastric juice miR-129 as a potential biomarker for screening gastric cancer. Med Oncol..

[46] Li Z, Lei H, Luo M, Wang Y, Dong L, Ma Y (2015). DNA methylation downregulated mir-10b acts as a tumor suppressor in gastric cancer. Gastric Cancer..

[47] Li T, Fan J, Wang B, Traugh N, Chen Q, Liu JS (2017). TIMER: a web server for comprehensive analysis of tumor-infiltrating immune cells. Cancer Res..

[48] Janky R, Verfaillie A, Imrichova H, Van de Sande B, Standaert L, Christiaens V (2014). iRegulon: from a gene list to a gene regulatory network using large motif and track collections. PLoS Comput Biol..

