# Supplementary material for: Identification and validation of methylated differentially expressed miRNAs and immune infiltrate profile in EBV-associated gastric cancer
Source: Clin Epigenetics. 2021 Jan 29;13:22. doi: 10.1186/s13148-020-00989-0 (PMC7845045; doi:10.1186/s13148-020-00989-0)
Supplement: Supplementary file 1 — Additional file 1: Figure S1. Venn Diagram showing the details of crossmatch of the multiple datasets. The display and analysis of genome methylation profile of GC and EBV cases. (a) Differentially expressed miRNAs of GC were rendered after the crossmatch of TCGA miRNA-seq and GSE87785, while differentially expressed methylated miRNAs were discovered using TCGA-methylation GC dataset. The EBV-related differentially expressed methylated miRNAs were analyzed using TCGA sequencing data. There was only one miRNA, miR-129-2-3p, that was simultaneously fit the GC and EBV criteria. (b) Differentially expressed genes (DEGs) were determined after crossmatching the GC datasets of TCGA RNA-seq and GSE66229 and the EBV datasets of TCGA RNA-seq and GSE51575. 30 DEGs were finally discovered. [file 13148_2020_989_MOESM1_ESM.docx]

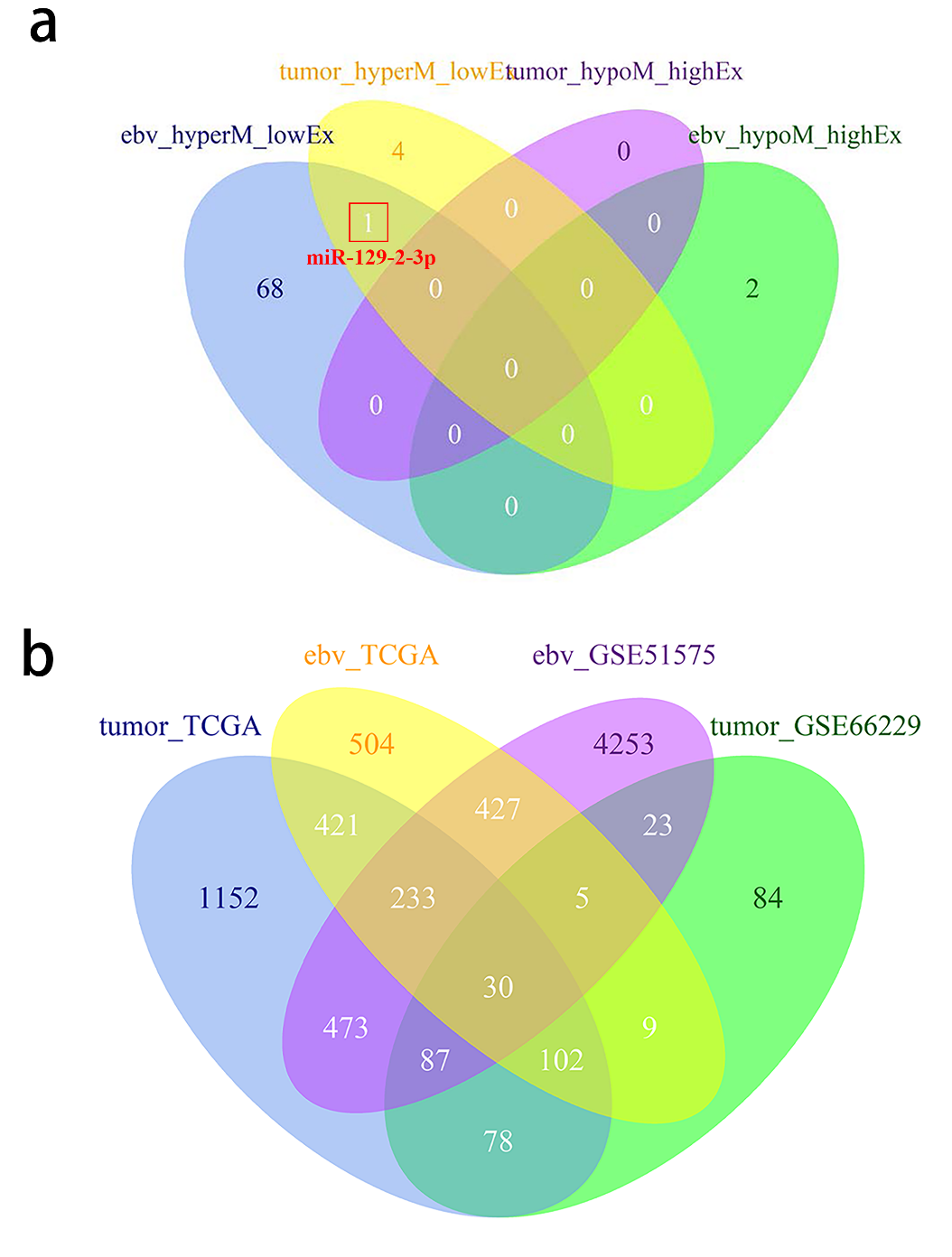


Fig. S1 Venn Diagram showing the details of crossmatch of the multiple datasets

The display and analysis of genome methylation profile of GC and EBV cases. (a) Differentially expressed miRNAs of GC were rendered after the crossmatch of TCGA miRNA-seq and GSE87785, while differentially expressed methylated miRNAs were discovered using TCGA-methylation GC dataset. The EBV-related differentially expressed methylated miRNAs were analyzed using TCGA sequencing data. There was only one miRNA, miR-129-2-3p, that was simultaneously fit the GC and EBV criteria. (b) Differentially expressed genes (DEGs) were determined after crossmatching the GC datasets of TCGA RNA-seq and GSE66229 and the EBV datasets of TCGA RNA-seq and GSE51575. 30 DEGs were finally discovered.
