# Supplementary material for: Identification and validation of methylated differentially expressed miRNAs and immune infiltrate profile in EBV-associated gastric cancer
Source: Clin Epigenetics. 2021 Jan 29;13:22. doi: 10.1186/s13148-020-00989-0 (PMC7845045; doi:10.1186/s13148-020-00989-0)
Supplement: Supplementary file 2 — Additional file 2: Figure S2. Circos plot depicting the genome-wide methylation profile of GC (a) and EBV (b) cases from the TCGA database. Hypermethylation is represented by red colors and hypomethylation by blue. From the inner circles to outer layer are hypomethylation peak plot, hypermethylation peak plot and methylation scatter plot. [file 13148_2020_989_MOESM2_ESM.docx]

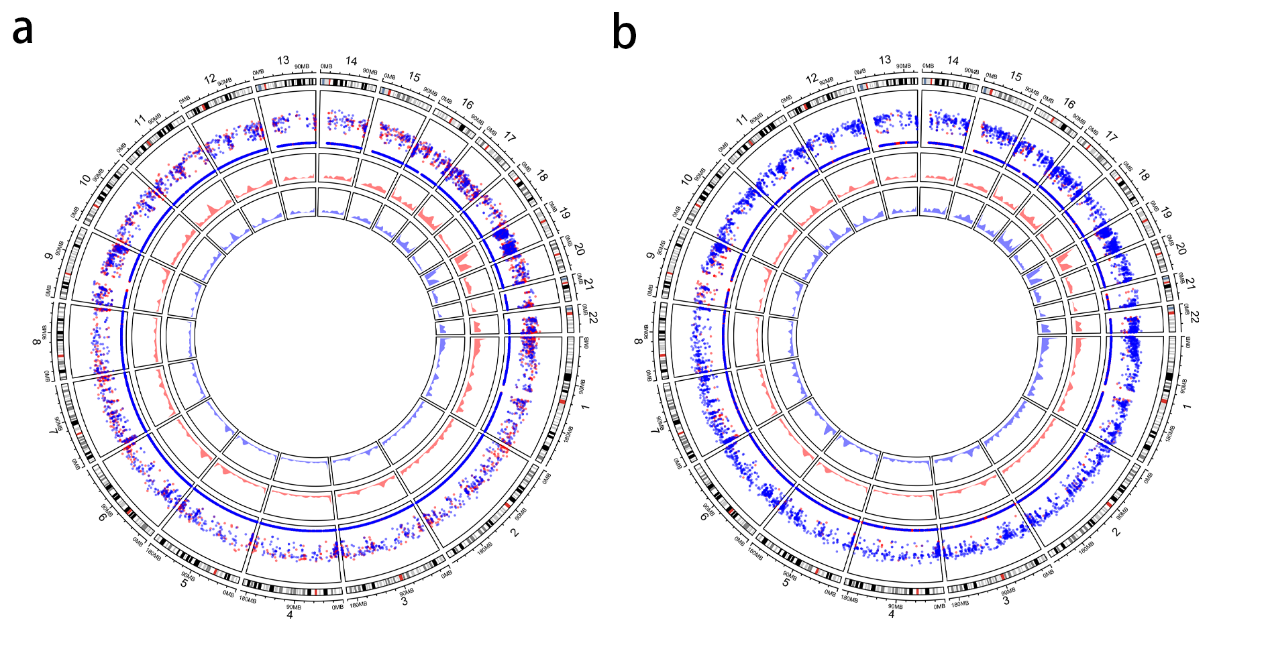


Fig. S2 Circos plot depicting the genome-wide methylation profile of GC (a) and EBV (b) cases from the TCGA database. Hypermethylation is represented by red colors and hypomethylation by blue. From the inner circles to outer layer are hypomethylation peak plot, hypermethylation peak plot and methylation scatter plot.
