# Supplementary material for: Identification and validation of methylated differentially expressed miRNAs and immune infiltrate profile in EBV-associated gastric cancer
Source: Clin Epigenetics. 2021 Jan 29;13:22. doi: 10.1186/s13148-020-00989-0 (PMC7845045; doi:10.1186/s13148-020-00989-0)
Supplement: Supplementary file 3 — Additional file 3: Differentially expressed genes due to methylation in EBVaGC. [file 13148_2020_989_MOESM3_ESM.docx]

TMEM100

CKB

SMTN

FHL1

RBPMS2

CAP2

GKN1

SCUBE2

KCNMB1

E2F7

MMRN1

HOXA10

GFRA1

SPP1

DCN

PLA2G7

TNFRSF11B

NOVA1

NRXN3

LARP6

HOXA13

IDO1

AKAP12

ANO5

SYNPO2

CCL20

HOMER2

OGN

ULBP2

SCG2
