# Supplementary material for: Identification and validation of methylated differentially expressed miRNAs and immune infiltrate profile in EBV-associated gastric cancer
Source: Clin Epigenetics. 2021 Jan 29;13:22. doi: 10.1186/s13148-020-00989-0 (PMC7845045; doi:10.1186/s13148-020-00989-0)
Supplement: Supplementary file 4 — Additional file 4: Figure S3. The time-dependent ROC curves for AKAP12, with the highest AUC of 0.612 at 60 and 65 months. Figure S4. Multivariate (up) and univariate (down) Cox-regression for AKAP12. Figure S5. The time-dependent ROC curves for DCN, with the highest AUC of 0.623 at 60 and 65 months. Figure S6. Multivariate (up) and univariate Cox-regression of DCN. Figure S7. The time-dependent ROC curves for LARP6, with the highest AUC of 0.601 from 75 months to 120 months. Figure S8. Multivariate (up) and univariate (down) Cox-regression of LARP6. Figure S9. The time-dependent ROC curves for NOVA1, with the highest AUC of 0.565 at 50 and 55 months. Figure S10. Multivariate (up) and univariate (down) Cox-regression of NOVA1. Figure S11. The time-dependent ROC curves for OGN, with the highest AUC of 0.609 at 5 months. Figure S12. Multivariate (up) and univariate Cox-regression of OGN. Figure S13. The time-dependent ROC curves of SCUBE2, with the highest AUC of 0.710 at 70 months. Figure S14. Multivariate (up) and univariate (down) Cox-regression of SCUBE2. Figure S15. The time-dependent ROC curves of HOXA10, with the highest AUC of 0.606 at 5 months. Figure S16. Multivariate (up) and univariate (down) Cox-regression of HOXA10. [file 13148_2020_989_MOESM4_ESM.docx]

Figure. S3 The time-dependent ROC curves for AKAP12, with the highest AUC of 0.612 at 60 and 65 months.


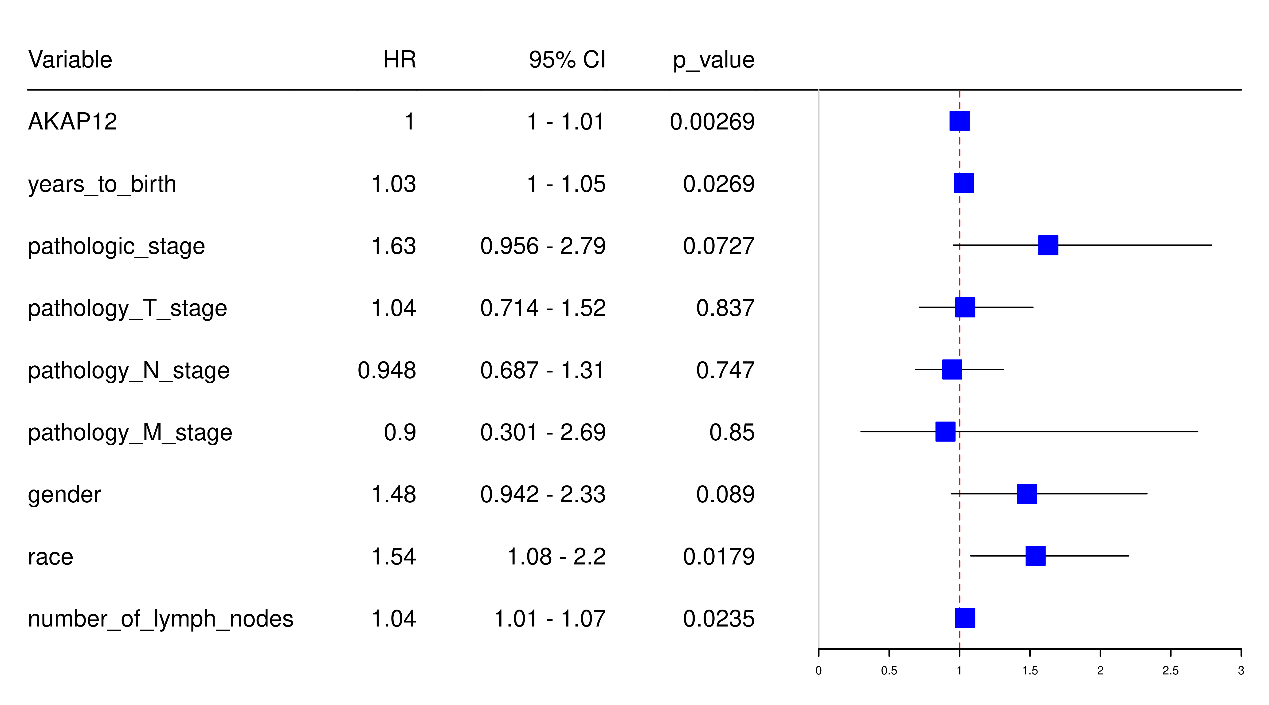


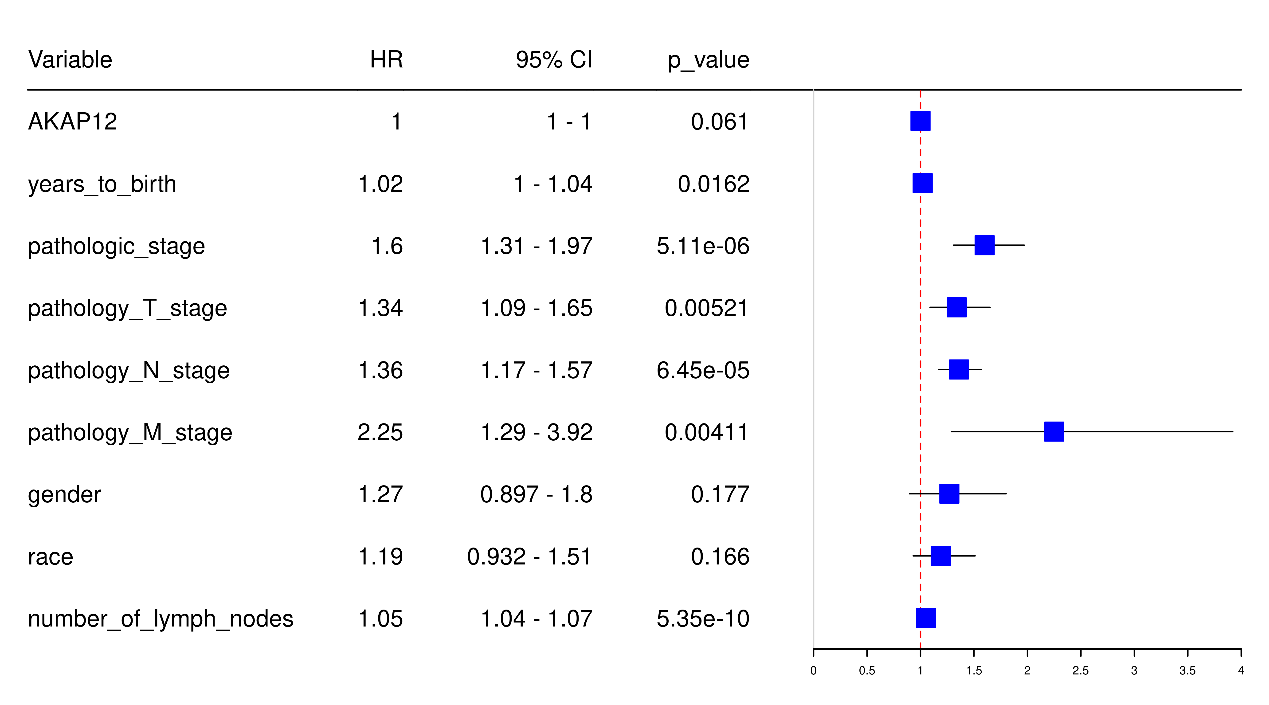


Figure. S4 Multivariate (up) and univariate (down) Cox-regression for AKAP12.





Figure. S5 The time-dependent ROC curves for DCN, with the highest AUC of 0.623 at 60 and 65 months.


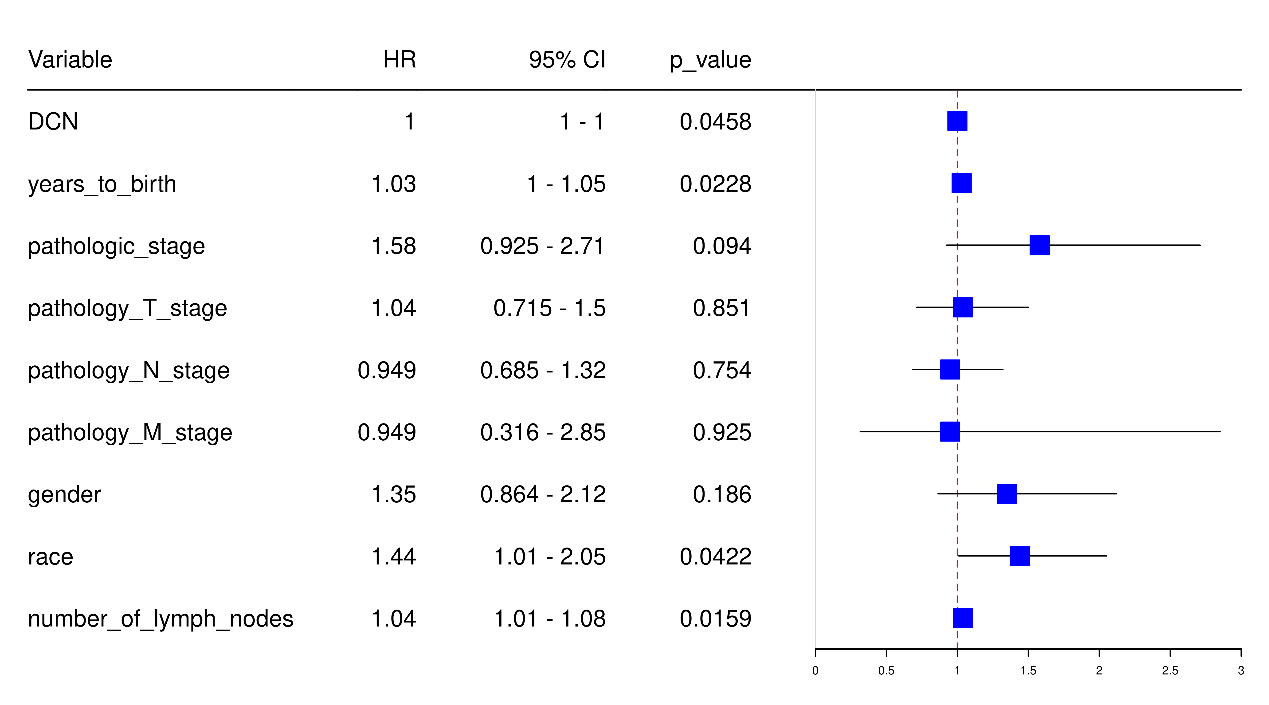

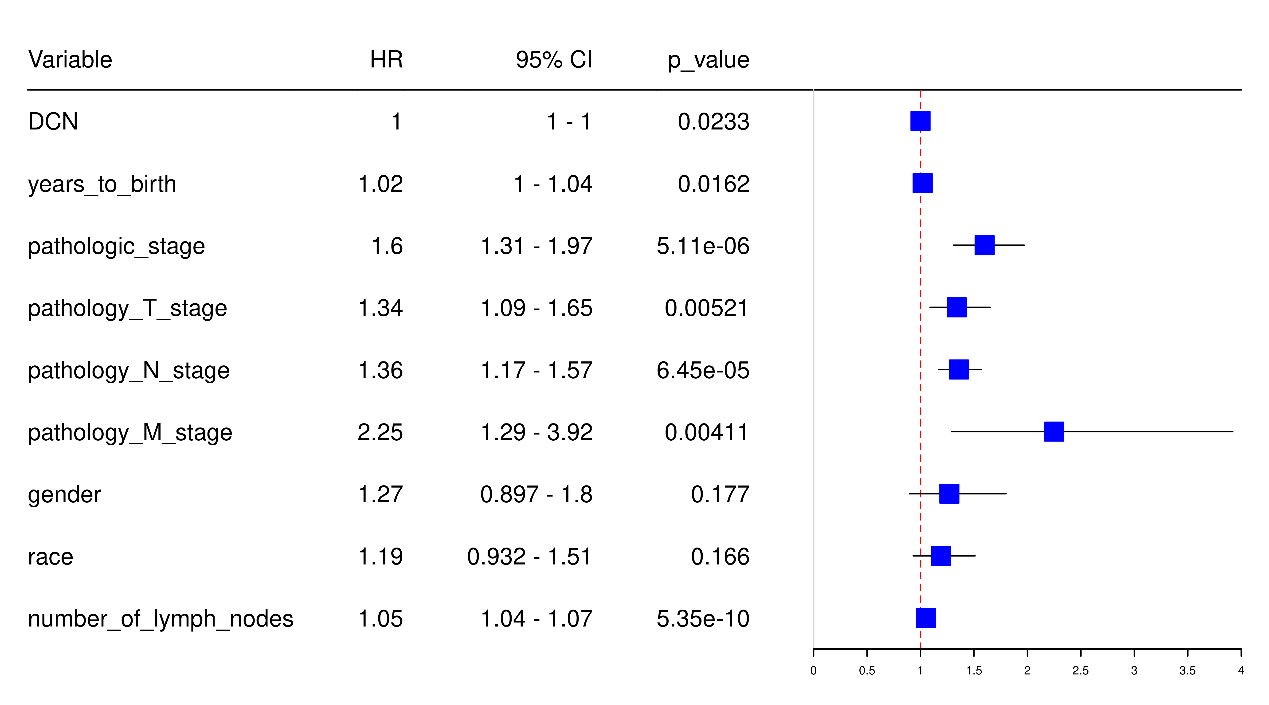


Figure. S6 Multivariate (up) and univariate Cox-regression of DCN.





Figure. S7 The time-dependent ROC curves for LARP6, with the highest AUC of 0.601 from 75 months to 120 months.


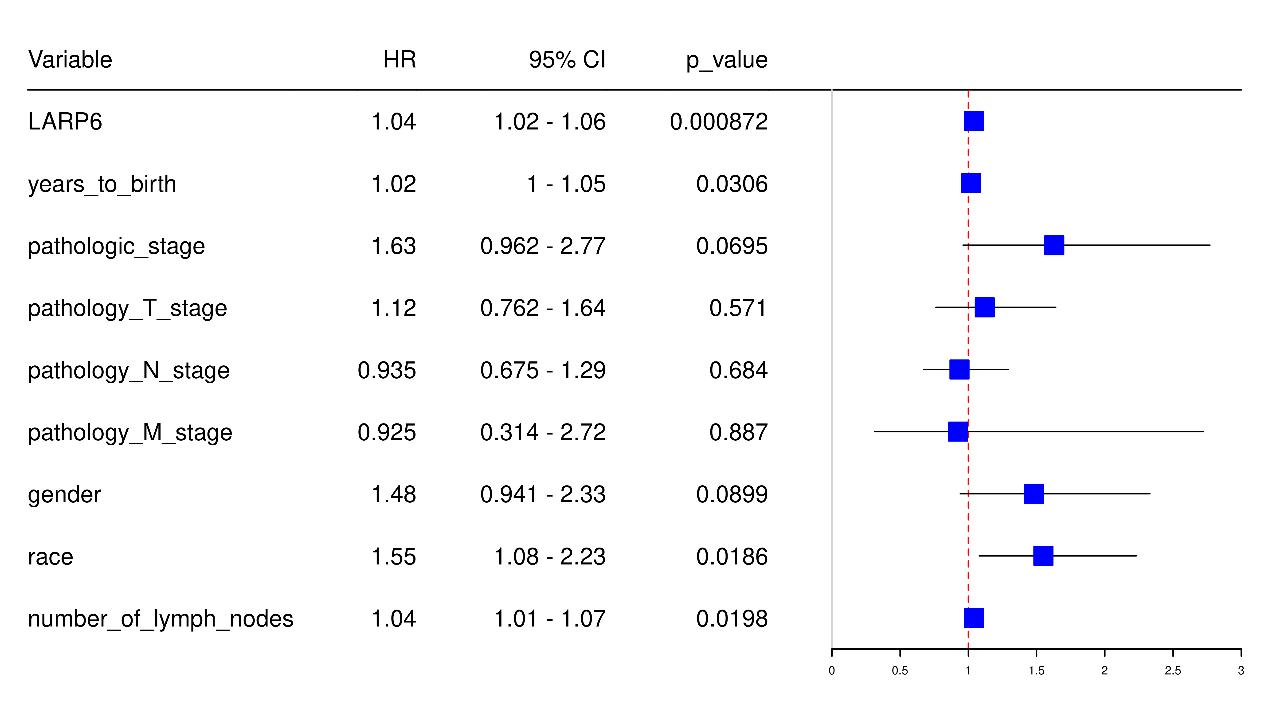

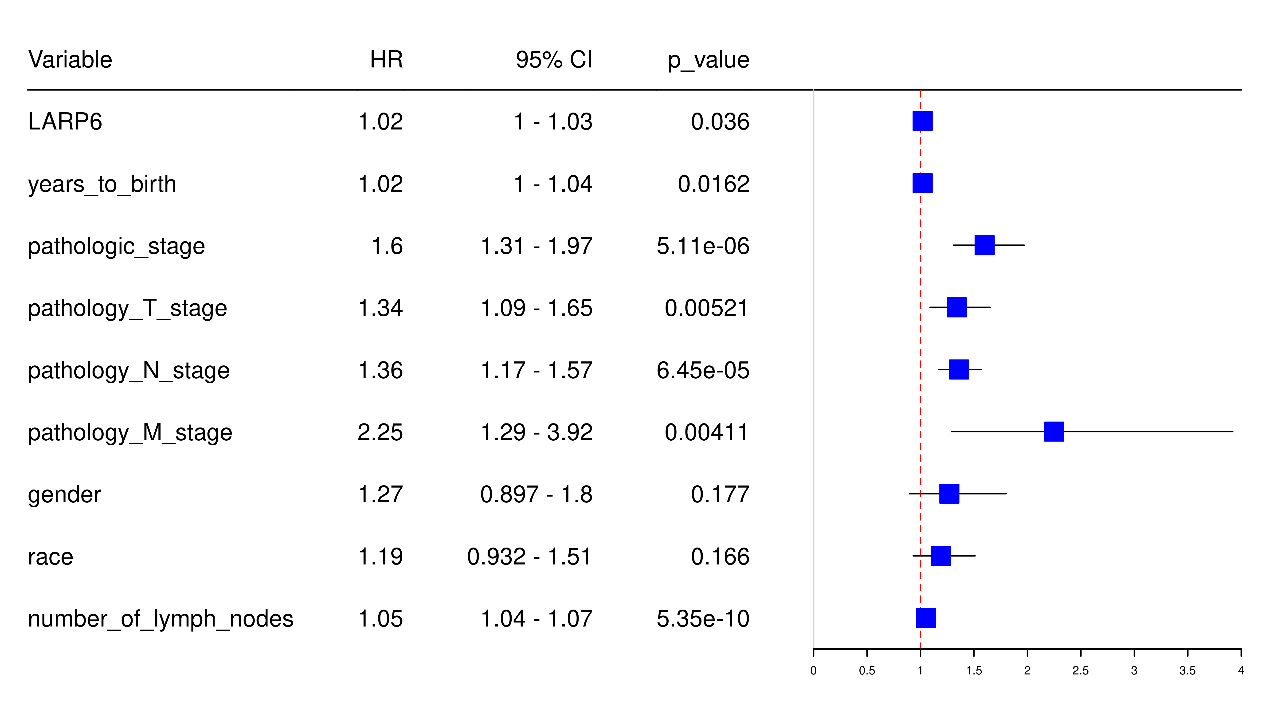


Figure. S8 Multivariate (up) and univariate (down) Cox-regression of LARP6.





Figure. S9 The time-dependent ROC curves for NOVA1, with the highest AUC of 0.565 at 50 and 55 months.


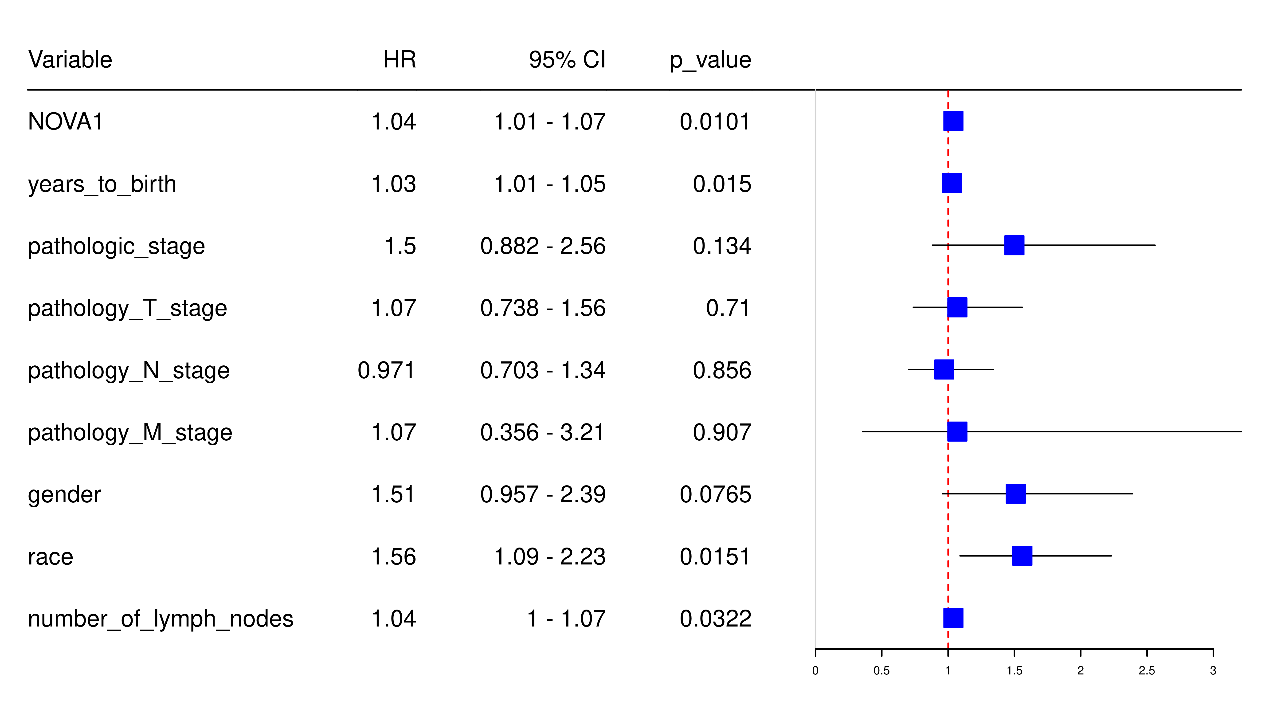

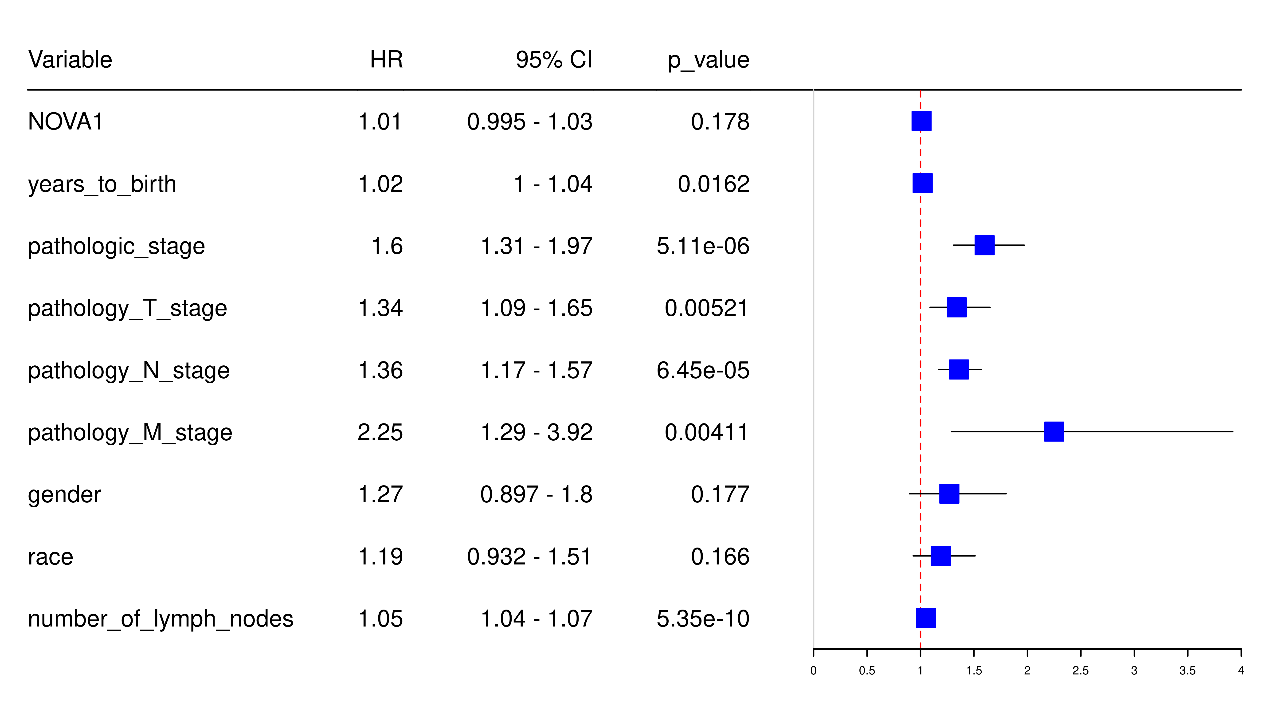


Figure. S10 Multivariate (up) and univariate (down) Cox-regression of NOVA1.





Figure. S11 The time-dependent ROC curves for OGN, with the highest AUC of 0.609 at 5 months.


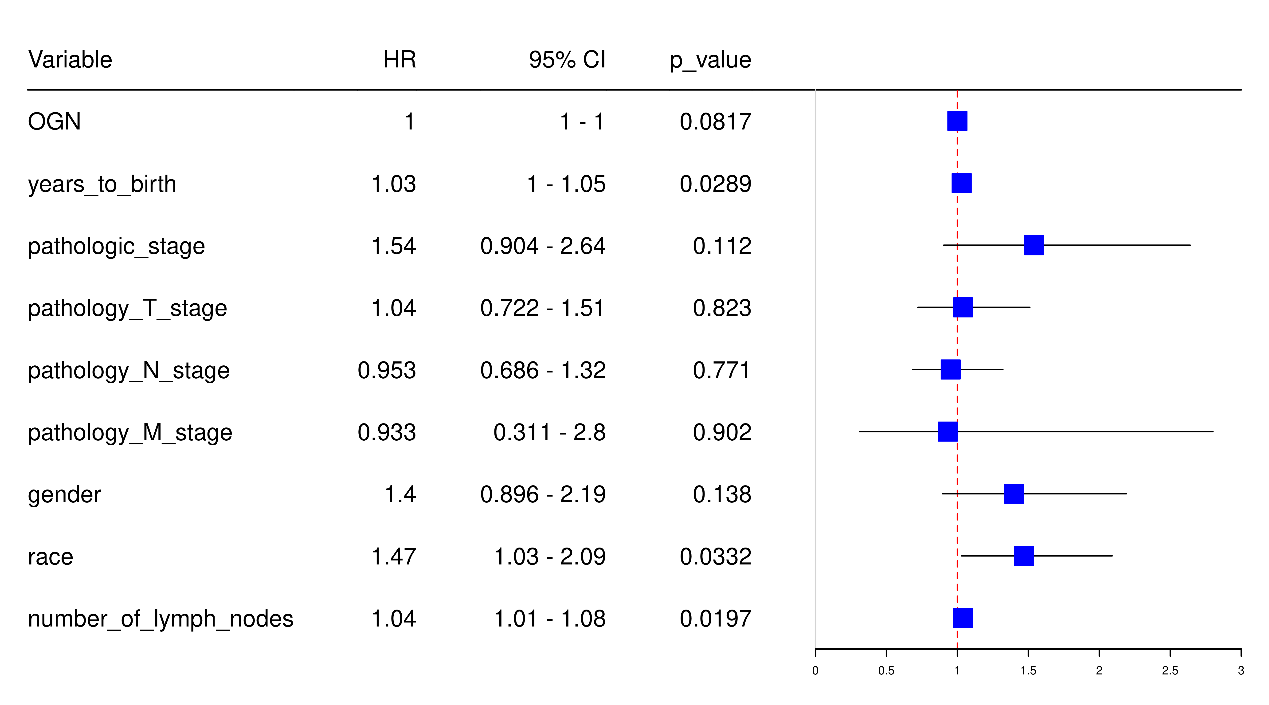

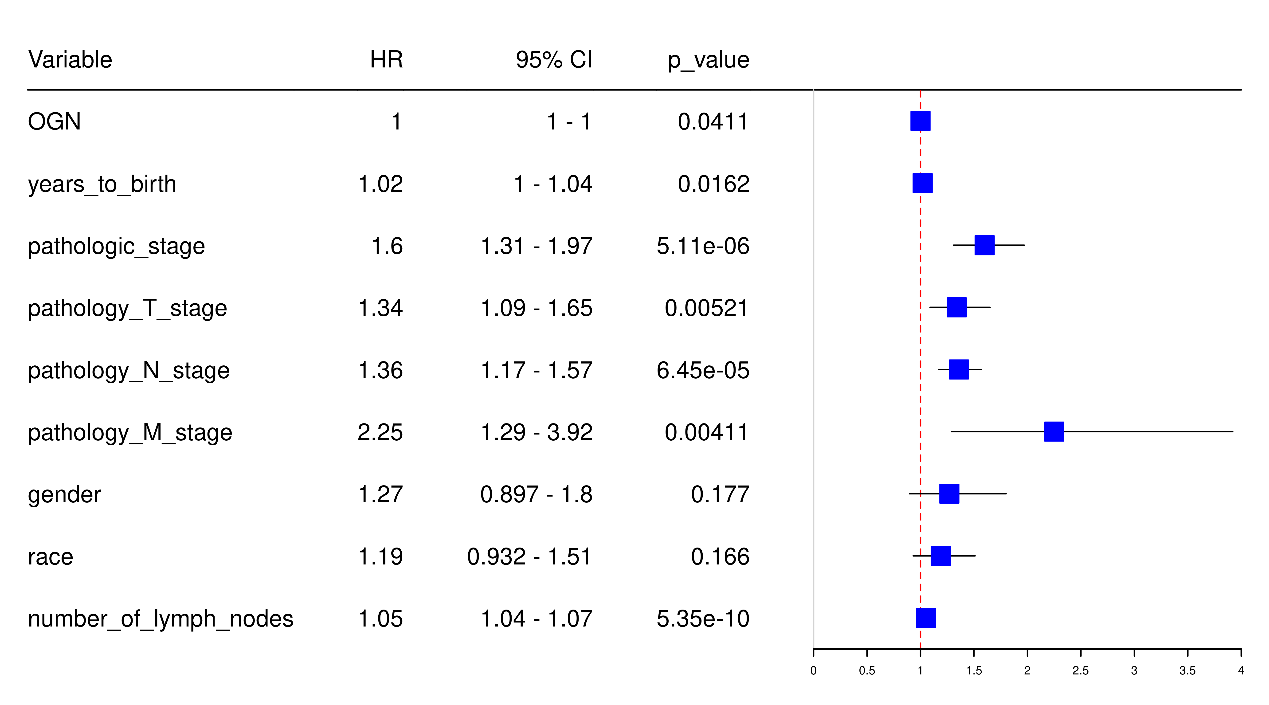


Figure. S12 Multivariate (up) and univariate Cox-regression of OGN.





Figure. S13 The time-dependent ROC curves of SCUBE2, with the highest AUC of 0.710 at 70 months.


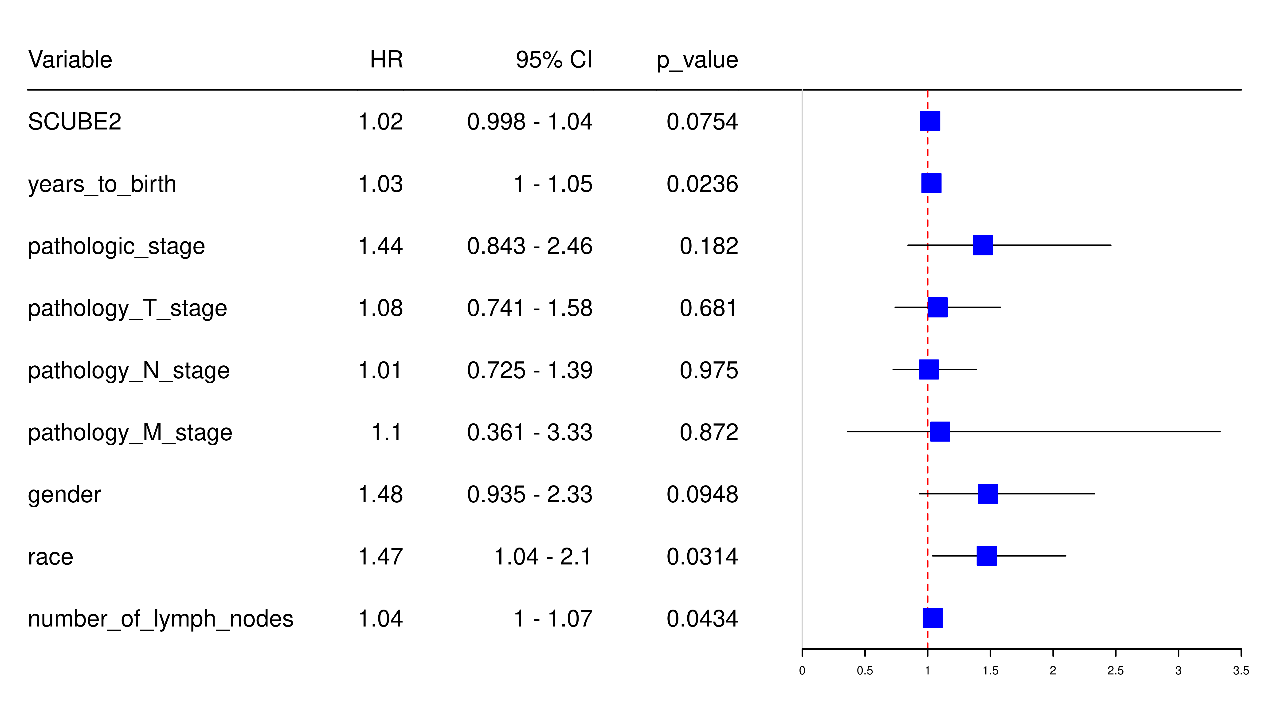

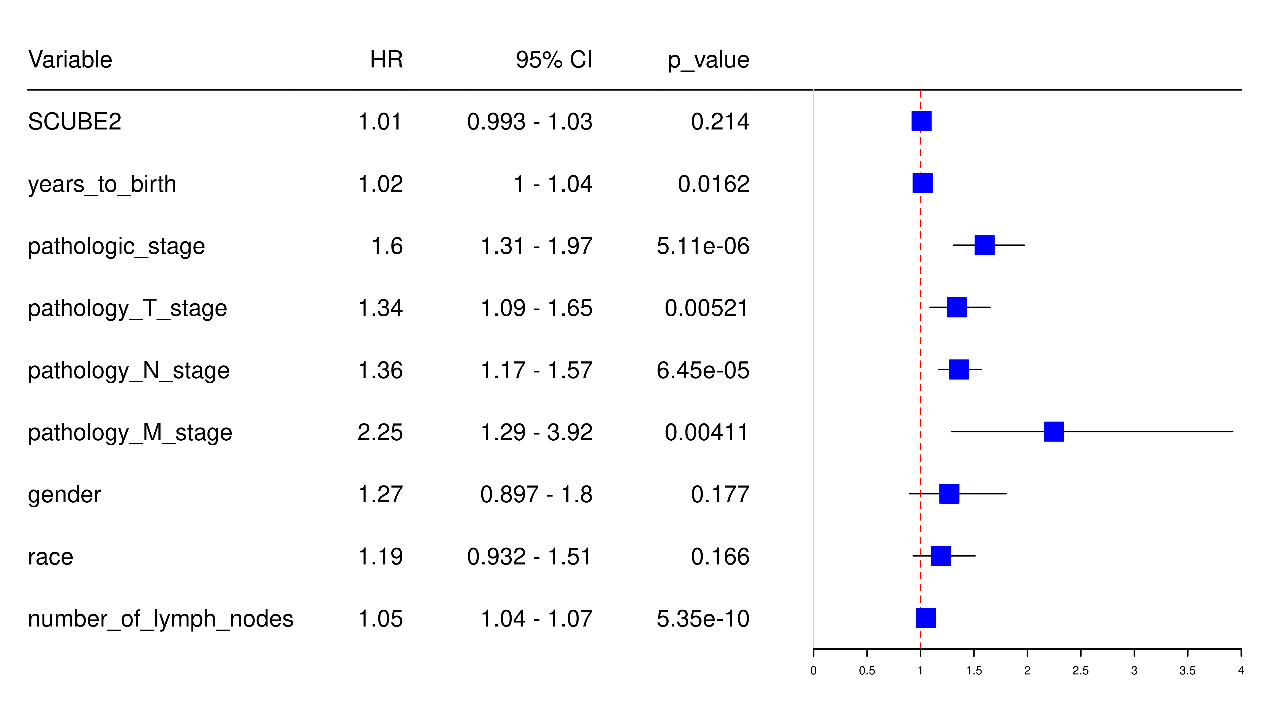


Figure. S14 Multivariate (up) and univariate (down) Cox-regression of SCUBE2.





Figure. S15 The time-dependent ROC curves of HOXA10, with the highest AUC of 0.606 at 5 months.


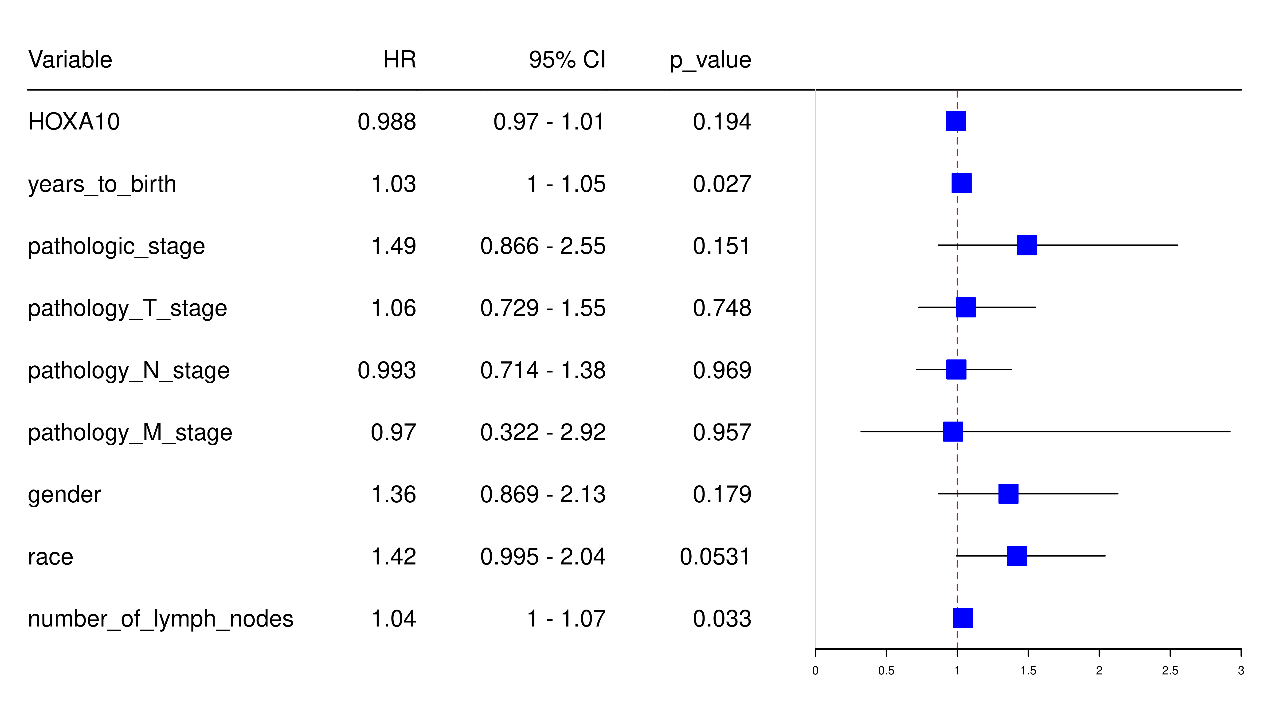

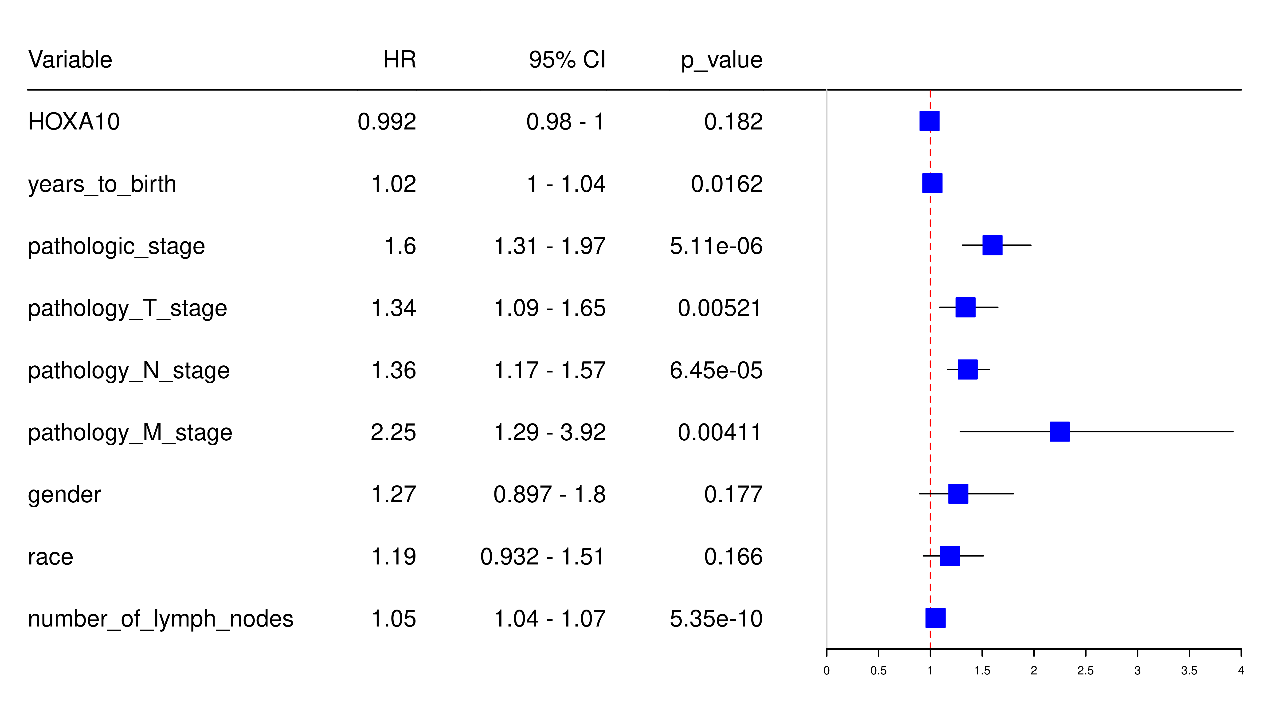


Figure. S16 Multivariate (up) and univariate (down) Cox-regression of HOXA10.
