# Supplementary material for: Identification and validation of methylated differentially expressed miRNAs and immune infiltrate profile in EBV-associated gastric cancer
Source: Clin Epigenetics. 2021 Jan 29;13:22. doi: 10.1186/s13148-020-00989-0 (PMC7845045; doi:10.1186/s13148-020-00989-0)
Supplement: Supplementary file 5 — Additional file 5: Figure S17. Tumor infiltrating immune cell analysis of the 7 hub genes. (a, b) AKAP12 and OGN were correlated with the enrichment of all the 6 types of TIICs, with particularly higher partial correlation coefficients in CD4+ T cell and macrophage. (c, d) DCN and HOXA10 were correlated with the enrichment of all the TIICs except B cell, with a particularly higher partial correlation coefficient in macrophage. (e) NOVA1 was correlated with all types of TIICs except neutrophil, with particularly higher partial correlation coefficients in CD4+ T cell and macrophage. (f) SCUBE2 was associated with the enrichment of B cell, CD4+ T cell, macrophage and dendritic cell, also with higher partial correlation coefficients in CD4+ T cell and macrophage. (g) LARP6 was associated with the enrichment of CD4+ T cell, macrophage and dendritic cell, with the highest partial correlation coefficient in macrophage. [file 13148_2020_989_MOESM5_ESM.docx]

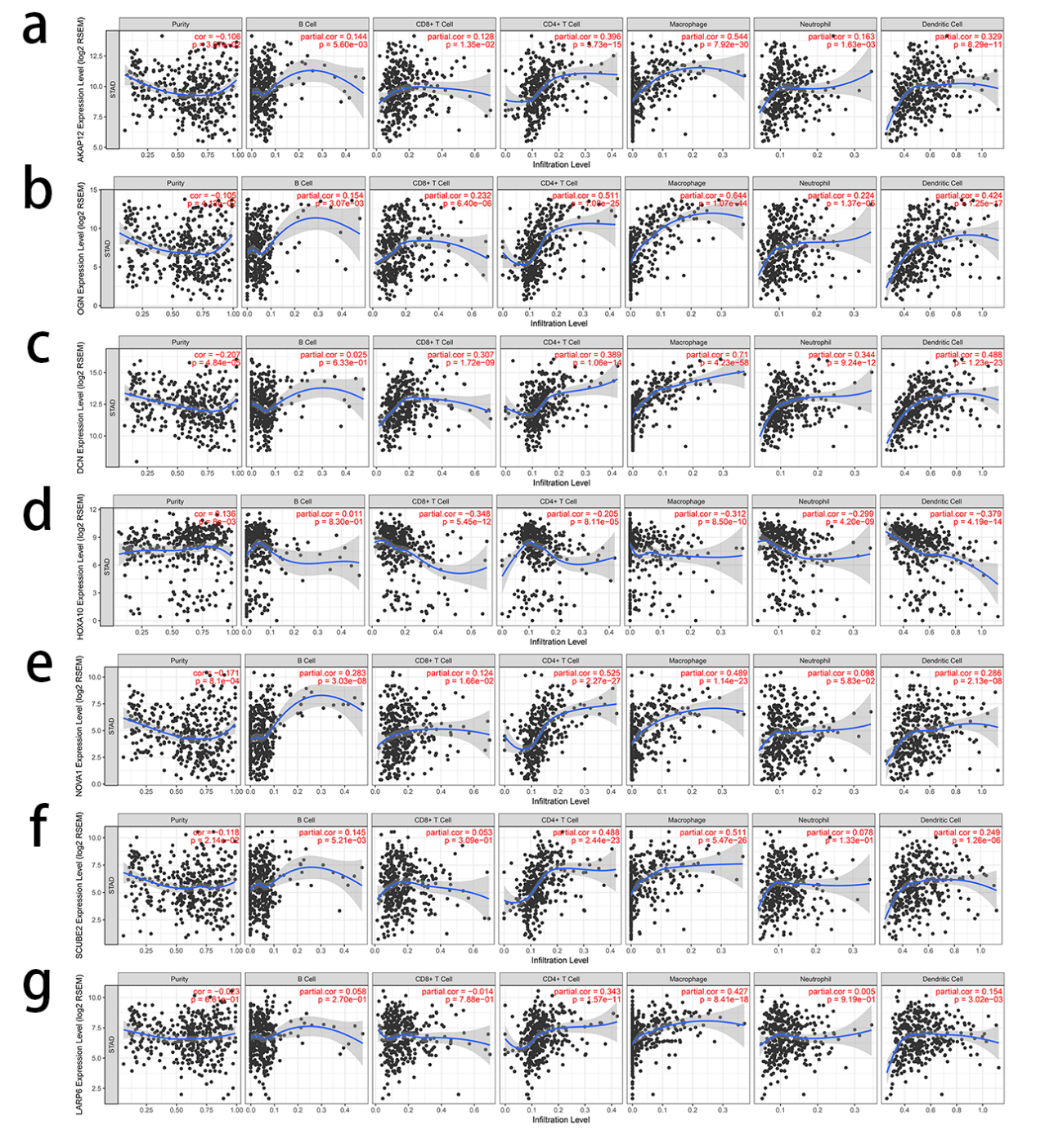


Fig. S17 Tumor infiltrating immune cell analysis of the 7 hub genes. (a, b) AKAP12 and OGN were correlated with the enrichment of all the 6 types of TIICs, with particularly higher partial correlation coefficients in CD4^+^ T cell and macrophage. (c, d) DCN and HOXA10 were correlated with the enrichment of all the TIICs except B cell, with a particularly higher partial correlation coefficient in macrophage. (e) NOVA1 was correlated with all types of TIICs except neutrophil, with particularly higher partial correlation coefficients in CD4^+^ T cell and macrophage. (f) SCUBE2 was associated with the enrichment of B cell, CD4^+^ T cell, macrophage and dendritic cell, also with higher partial correlation coefficients in CD4^+^ T cell and macrophage. (g) LARP6 was associated with the enrichment of CD4^+^ T cell, macrophage and dendritic cell, with the highest partial correlation coefficient in macrophage.
