# Supplementary material for: Identification and validation of methylated differentially expressed miRNAs and immune infiltrate profile in EBV-associated gastric cancer
Source: Clin Epigenetics. 2021 Jan 29;13:22. doi: 10.1186/s13148-020-00989-0 (PMC7845045; doi:10.1186/s13148-020-00989-0)
Supplement: Supplementary file 6 — Additional file 6: Table S1. Primers for QPCR assays. Figure S18. The relative expression of miR-129-2-3p of different GC cell lines transfected with inhibitor/mimics. **P=0.0013, ****P<0.0001. [file 13148_2020_989_MOESM6_ESM.docx]

| Primers for U6 |  |
| --- | --- |
| Forward | CTCGCTTCGGCAGCACATATACT |
| Reverse | ACGCTTCACGAATTTGCGTGTC |
| Primers for hsa-miR-129-2-3p |  |
| RT | CTCAACTGGTGTCGTGGAGTCGGCAATTCAGTTGAGATGCTT |
| Forward | ACACTCCAGCTGGGAAGCCCTTACCCCAAA |
| Reverse | TGGTGTCGTGGAGTCG |

Table. S1 Primers for QPCR assays.


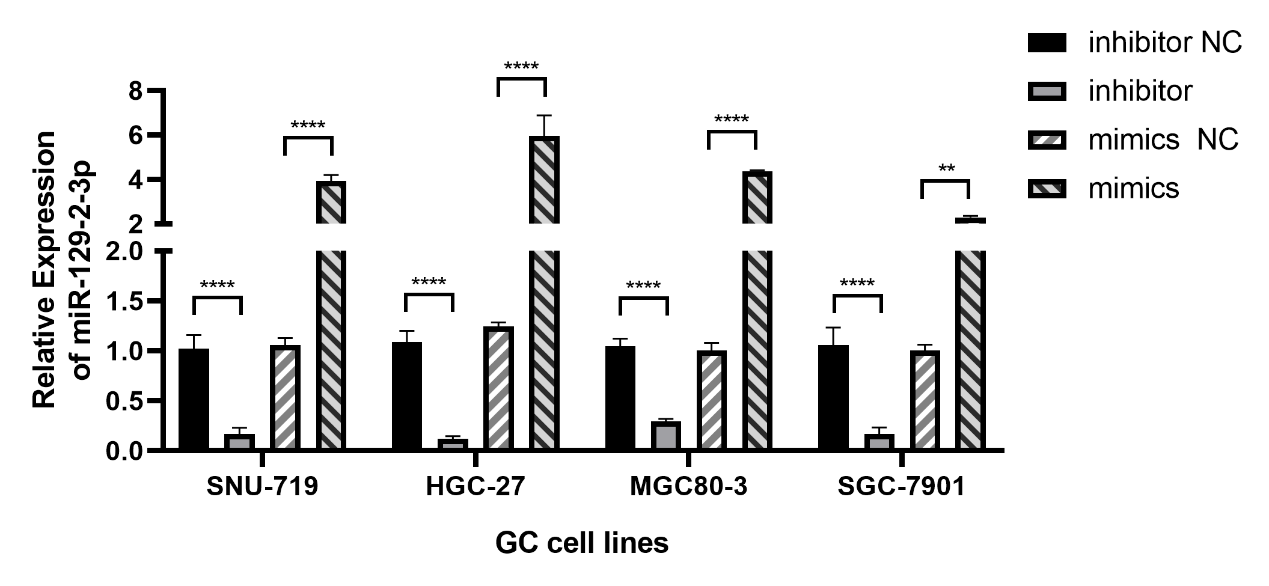


Fig. S18 The relative expression of miR-129-2-3p of different GC cell lines transfected with inhibitor/mimics. ^**^ *P*=0.0013, ^****^ *P*<0.0001
