# Supplementary material for: Identification and validation of methylated differentially expressed miRNAs and immune infiltrate profile in EBV-associated gastric cancer
Source: Clin Epigenetics. 2021 Jan 29;13:22. doi: 10.1186/s13148-020-00989-0 (PMC7845045; doi:10.1186/s13148-020-00989-0)
Supplement: Supplementary file 7 — Additional file 7: Figure S19. Bisulfite Sequencing PCR (BSP) to test the methylation level of the EBVaGC cell line SNU-719 and the EBVnGC cell lines MGC80-3, HGC-27 and SGC-7901. Group1, MGC80-3, HGC-27 and SGC-7901; Group 2, SNU-719. (a) There are 4 regions rich in methylation, 404-510bp, 1026-1211bp, 1700-1914bp and 1949-2054bp from the TSS of miR-129-2-3p. (b) Regions 1 and 4 in SNU-719 are significantly higher methylated than its EBVnGC counterpart. [file 13148_2020_989_MOESM7_ESM.docx]

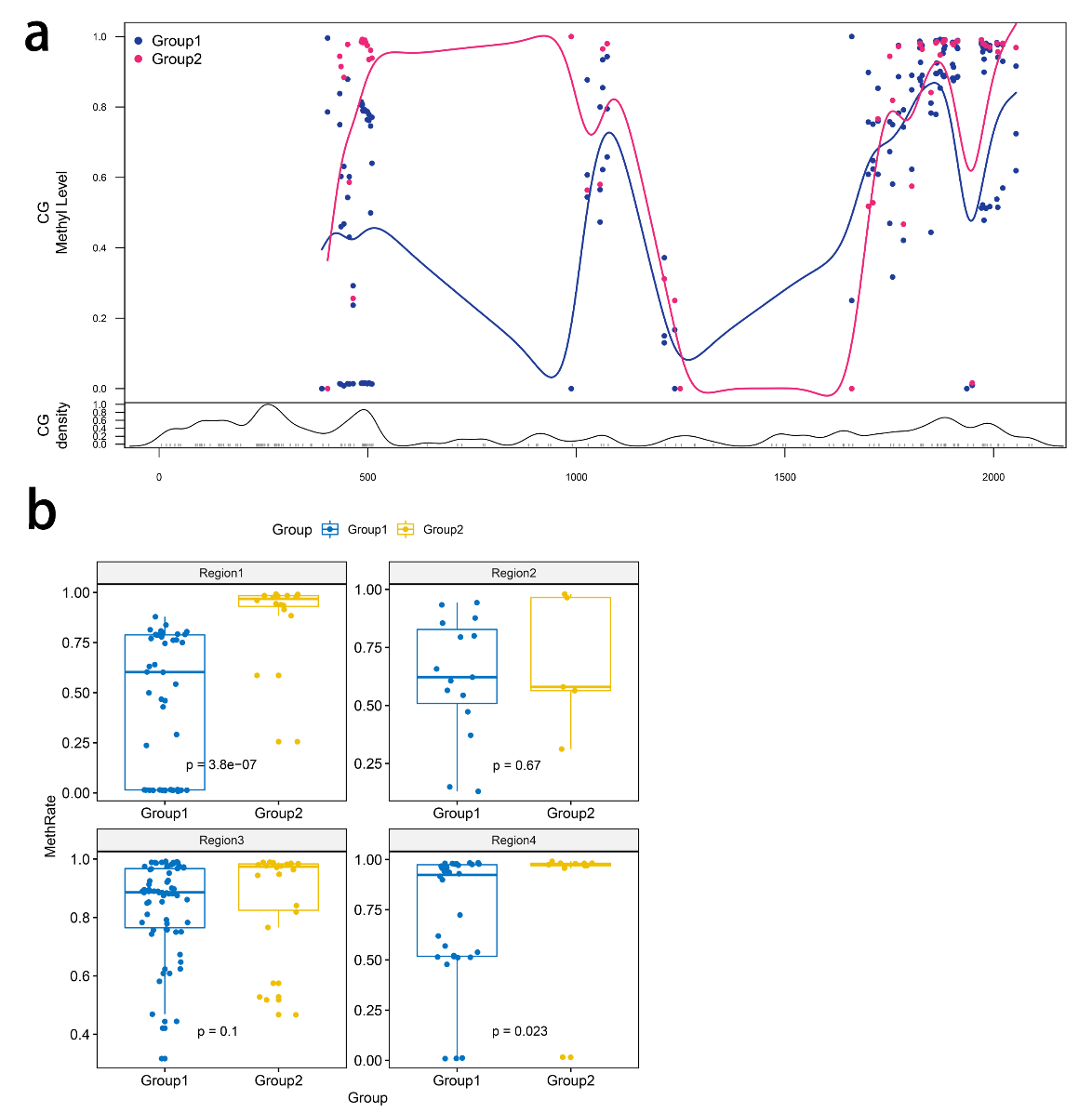


Fig. S19 Bisulfite Sequencing PCR (BSP) to test the methylation level of the EBVaGC cell line SNU-719 and the EBVnGC cell lines MGC80-3, HGC-27 and SGC-7901. Group1, MGC80-3, HGC-27 and SGC-7901; Group 2, SNU-719. (a) There are 4 regions rich in methylation, 404-510bp, 1026-1211bp, 1700-1914bp and 1949-2054bp from the TSS of miR-129-2-3p. (b) Regions 1 and 4 in SNU-719 are significantly higher methylated than its EBVnGC counterpart.
