# Supplementary figures and images for: Identification and validation of methylated differentially expressed miRNAs and immune infiltrate profile in EBV-associated gastric cancer
Source: Clin Epigenetics. 2021 Jan 29;13:22. doi: 10.1186/s13148-020-00989-0 (PMC7845045; doi:10.1186/s13148-020-00989-0)

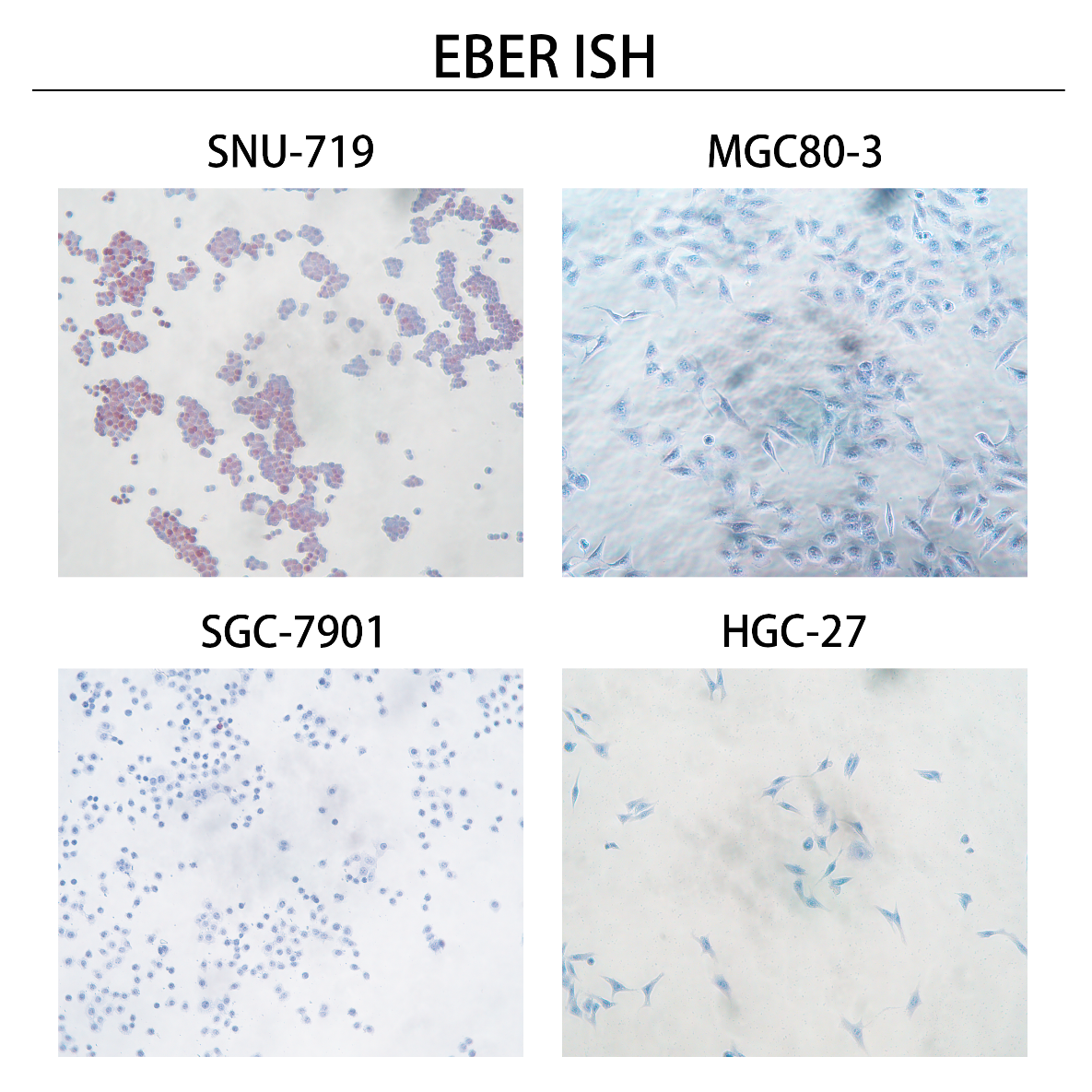


Fig. S20 in situ hybridization (ISH) assay using EBER probe to detect EBV infection in GC cells

Supplement: Supplementary file 8 — Additional file 8: Figure S20. In situ hybridization (ISH) assay using EBER probe to detect EBV infection in GC cells. [file 13148_2020_989_MOESM8_ESM.docx]

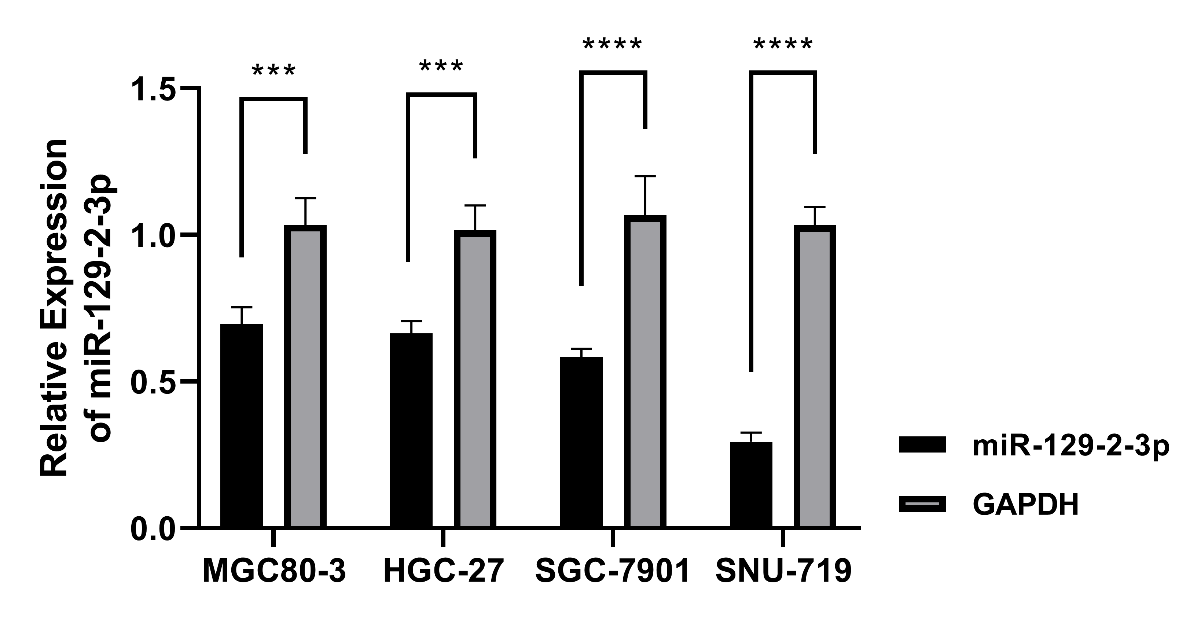


Fig. S21 the endogenous expression of miR-129-2-3p in different GC cell lines

Supplement: Supplementary file 9 — Additional file 9: Figure S21. The endogenous expression of miR-129-2-3p in different GC cell lines. [file 13148_2020_989_MOESM9_ESM.docx]

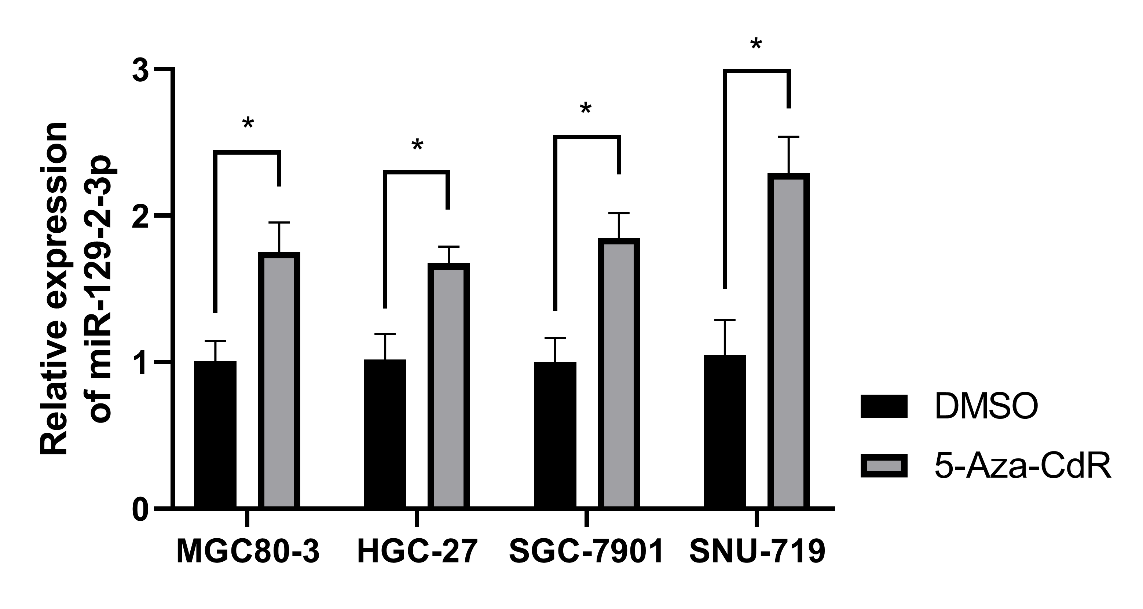


Fig. S22 Expression changes in miR-129-2-3p in the GC cell lines by the treatment of demethylator 5-Aza-CdR.

Supplement: Supplementary file 10 — Additional file 10: Figure S22. Expression changes in miR-129-2-3p in the GC cell lines by the treatment of demethylator 5-Aza-CdR. [file 13148_2020_989_MOESM10_ESM.docx]

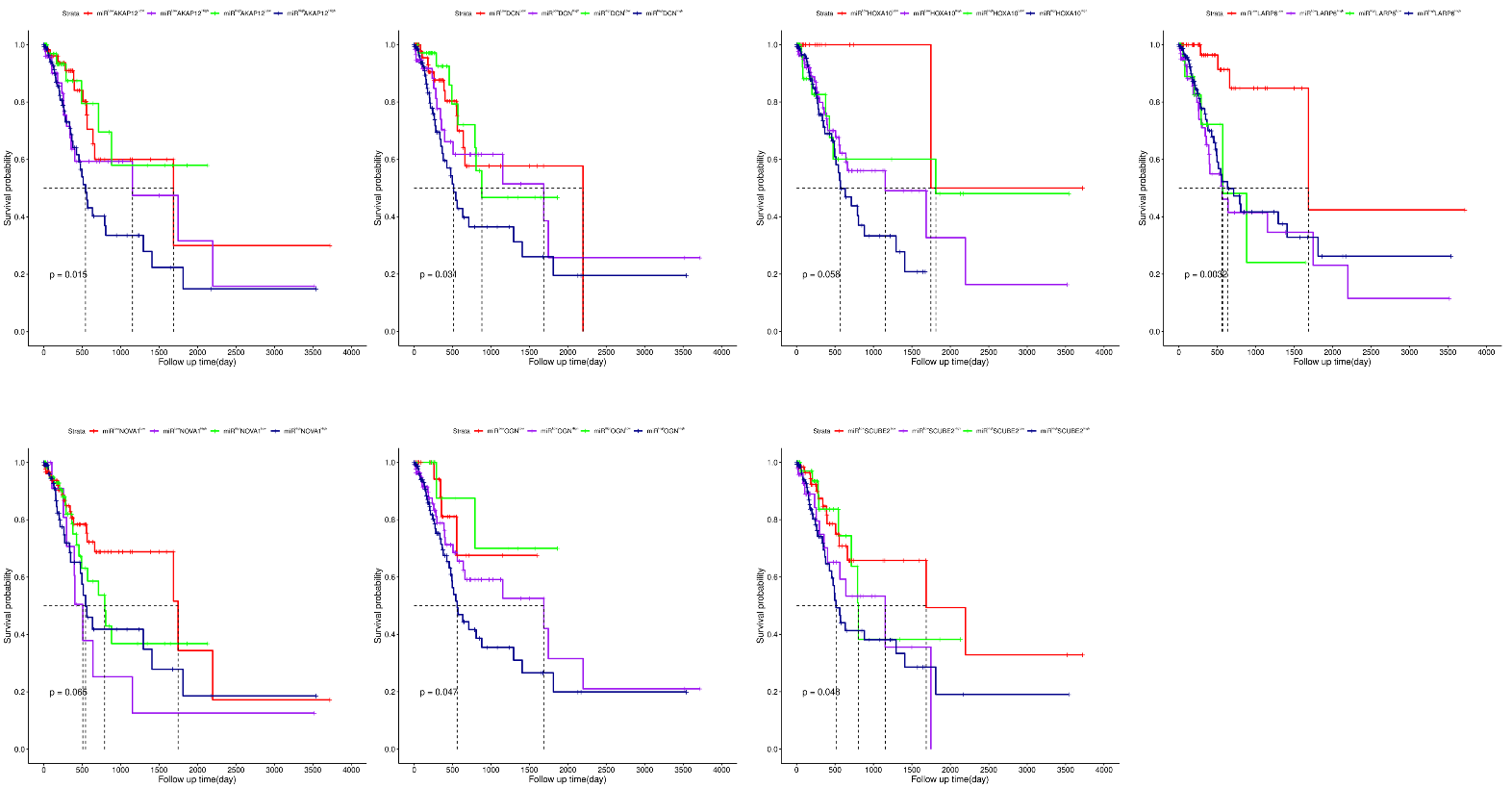


Fig. S25 Survival analysis of the target genes combining miR-129-2-3p

Supplement: Supplementary file 13 — Additional file 13: Figure S25. Survival analysis of the target genes combining miR-129-2-3p. [file 13148_2020_989_MOESM13_ESM.docx]

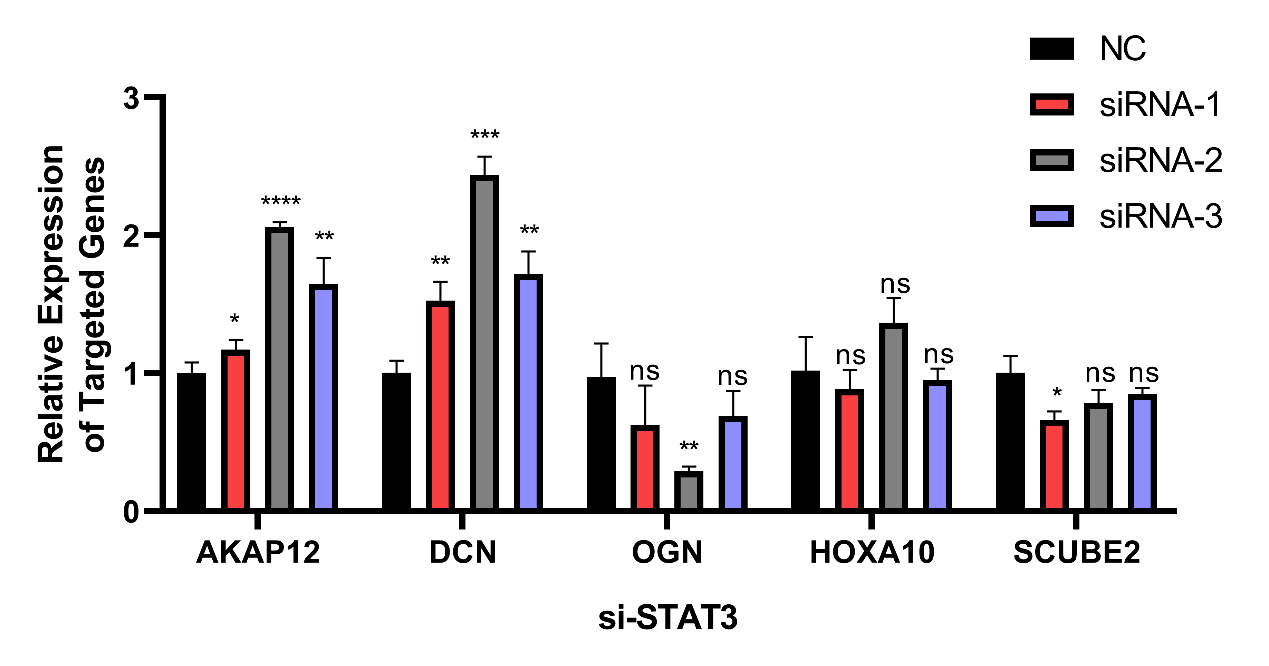


Fig. S26 The expression changes of the putative target genes transfected with siRNAs of STAT3

Supplement: Supplementary file 14 — Additional file 14: Figure S26. The expression changes of the putative target genes transfected with siRNAs of STAT3. [file 13148_2020_989_MOESM14_ESM.docx]
