# Supplementary material for: Identification and validation of methylated differentially expressed miRNAs and immune infiltrate profile in EBV-associated gastric cancer
Source: Clin Epigenetics. 2021 Jan 29;13:22. doi: 10.1186/s13148-020-00989-0 (PMC7845045; doi:10.1186/s13148-020-00989-0)
Supplement: Supplementary file 11 — Additional file 11: Figure S23. Dual Luciferase Reporting system to validate the binding activity of miR-129-2-3p to the 3’-UTR of target genes. [file 13148_2020_989_MOESM11_ESM.docx]

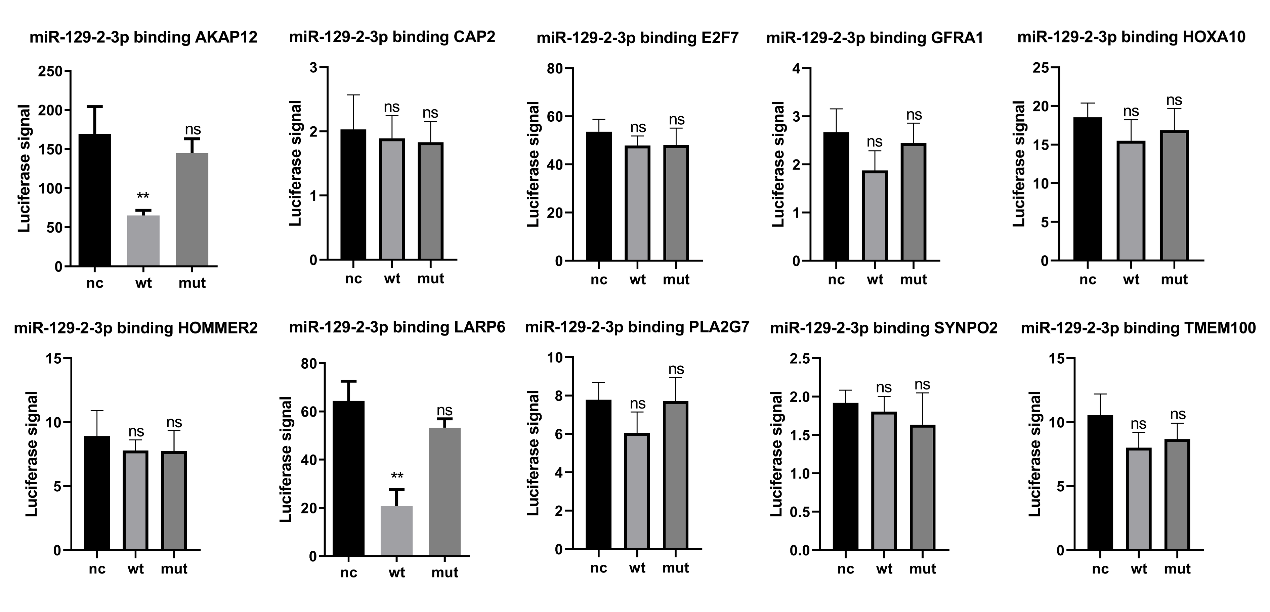


Fig. S23 Dual Luciferase Reporting system to validate the binding activity of miR-129-2-3p to the 3’-UTR of target genes
