# Supplementary material for: Identification and validation of methylated differentially expressed miRNAs and immune infiltrate profile in EBV-associated gastric cancer
Source: Clin Epigenetics. 2021 Jan 29;13:22. doi: 10.1186/s13148-020-00989-0 (PMC7845045; doi:10.1186/s13148-020-00989-0)
Supplement: Supplementary file 12 — Additional file 12: Figure S24. The expression changes of the putative target genes transfected with mimics or inhibitor of miR-129-2-3p. [file 13148_2020_989_MOESM12_ESM.docx]

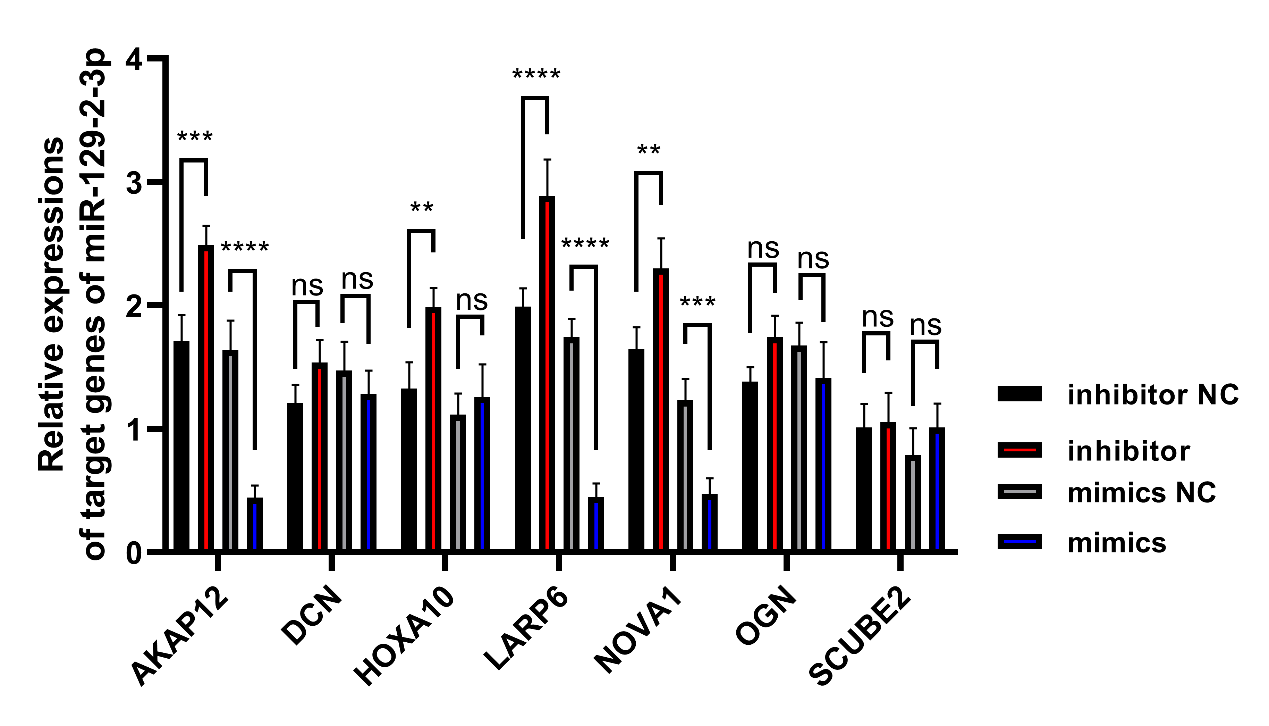


Fig. S24 The expression changes of the putative target genes transfected with mimics or inhibitor of miR-129-2-3p
